# Microtubule disruption synergizes with STING signaling to show potent and broad-spectrum antiviral activity

**DOI:** 10.1371/journal.ppat.1012048

**Published:** 2024-02-26

**Authors:** Jing Han, Zhimeng Wang, Fangping Han, Bo Peng, Juanjuan Du, Conggang Zhang

**Affiliations:** 1 State Key Laboratory of Membrane Biology, School of Pharmaceutical Sciences, Tsinghua University, Beijing, China; 2 Tsinghua-Peking Center for Life Sciences, Beijing, China; 3 Department of Microbiology Laboratory, Shenzhen Center for Disease Control and Prevention, Shenzhen, China; 4 MOE Key Laboratory of Bioorganic Phosphorus Chemistry &Chemical Biology, Tsinghua University Beijing, China; University of Southern California, UNITED STATES

## Abstract

The activation of stimulator of interferon genes (STING) signaling induces the production of type I interferons (IFNs), which play critical roles in protective innate immunity for the host to defend against viral infections. Therefore, achieving sustained or enhanced STING activation could become an antiviral immune strategy with potential broad-spectrum activities. Here, we discovered that various clinically used microtubule-destabilizing agents (MDAs) for the treatment of cancer showed a synergistic effect with the activation of STING signaling in innate immune response. The combination of a STING agonist cGAMP and a microtubule depolymerizer MMAE boosted the activation of STING innate immune response and showed broad-spectrum antiviral activity against multiple families of viruses. Mechanistically, MMAE not only disrupted the microtubule network, but also switched the cGAMP-mediated STING trafficking pattern and changed the distribution of Golgi apparatus and STING puncta. The combination of cGAMP and MMAE promoted the oligomerization of STING and downstream signaling cascades. Importantly, the cGAMP plus MMAE treatment increased STING-mediated production of IFNs and other antiviral cytokines to inhibit viral propagation *in vitro* and *in vivo*. This study revealed a novel role of the microtubule destabilizer in antiviral immune responses and provides a previously unexploited strategy based on STING-induced innate antiviral immunity.

## Author summary

Activation of innate immune signaling pathways provide potential to induce potent and broad-spectrum antiviral immune response. Meanwhile, modulation of the microtubule network may also disturb the normal replication cycle of viruses to serve as antiviral strategy. It is interesting to define whether the combination of the agonists of innate immunity and MDAs could be used in the treatment of viral infection. In this study, we show that MDAs synergize with cGAMP-mediated STING signaling pathway to show broad-spectrum antiviral activity. We reveal that MMAE, a clinically used MDA in tumor treatment, disrupts microtubule network and alters STING trafficking signaling. The MMAE and cGAMP co-treatment enhances the TBK1-STING-IRF3 cascade, type I IFNs induction, and NF-kB immune response of the STING signaling. Importantly, the combination of MMAE and cGAMP triggers promising antiviral effect *in vitro* and *in vivo* in a STING-dependent manner. Thus, these findings could help inform future antiviral drugs by modulating cGAS-STING DNA sensing signaling and microtubule network.

## Introduction

Over the course of human history, viruses have posed substantial challenges to human health. In the combat against viral infections, drugs targeting viral components such as viral proteases have been successfully developed and widely used in clinic. Meanwhile, antibodies have been developed to block interactions between viruses and host cells. However, the effectiveness of these strategies quickly dwindled due to mutations in viral genes and subsequent drug resistance. To deal with the continuous challenge of viral infection, more potent and safer antiviral drugs exploiting host immune system would offer potential advantages including broad-spectrum activities and a lower likelihood to develop drug resistance [1,2].

Innate immunity, which is the first line of host immune system against the invasion of both DNA and RNA viruses, is rapidly activated upon detection of viral components, including proteins, DNAs, or RNAs. Growing evidence shows that cGAS (cyclic GMP-AMP synthetase)-STING (stimulator of interferon genes) signaling pathway plays a crucial role in sensing and responding to invasions from DNA viruses and retroviruses [3]. Upon binding to cytosolic DNAs, cGAS is activated and catalyzes the production of the second messenger, cGAMP [4]. Subsequently, cGAMP binds and activates STING, which recruits and triggers the activation of Tank binding kinase (TBK1). TBK1 phosphorylates the transcriptional factor IRF3, resulting in its translocation into nucleus and inducing robust type I IFNs response and NF-kB immune response [5,6]. The IFNs induce the expression of hundreds of interferon-stimulated genes (ISGs) in an autocrine and paracrine manner, and ISGs interfere with almost every step in the virus life cycle. Therefore, strategies to modulate STING signaling network and enhance STING-associated immune responses may have potential applications in antiviral therapies. Recently, STING agonists, both cyclic dinucleotides (CDN) and non-CDN agonists, have been developed [7,8] and demonstrated their therapeutic benefit by providing robust protection against different viruses [9–14], supporting STING as a novel antiviral target.

Studies have shown that the trafficking of STING is dynamically regulated by vesicle trafficking system [15,16]. STING is an ER-localized transmembrane protein and senses CDN ligands, such as 2’3’-cGAMP [17], 3’3’-cGAMP, c-di-AMP, and c-di-GMP [18]. Upon ligand binding, STING forms oligomers [5] and is transported from ER to ER-Golgi intermediate compartments (ERGIC) and to Golgi apparatus via trafficking pathway [19,20]. STING activates the downstream kinase TBK1 in Golgi and STING vesicles then exit from trans-Golgi network (TGN) and continue their journey to autophagosomes, endosomes, and lysosomes, where STING is ultimately degraded and the signal is terminated [21,22]. Therefore, perturbing STING trafficking may provide an alternative or additional approach in STING modulation. Mounting evidence indicate that microtubules, an essential component of the cytoskeleton, play a crucial role in intracellular trafficking. So, disruption of the microtubule network not only seriously affects the viral replication cycle, such as viral entry, intracellular transport, and cell-to-cell spread [23,24], but also affects the proper distribution and function of organelles and vesicles, including the trafficking of STING [25]. Importantly, we found that a microtubule-destabilizing agent (MDA), podofilox, enhanced STING signaling with antitumor activity in our previous studies [26]. We were intrigued to hypothesize that MDAs may have antiviral activity by increasing STING signaling and innate immunity. It was reported that MDAs, such as nocodazole, colchicine, and vinblastine, can inhibit viral infection in cells at micromolar concentrations [27,28], while the antiviral mechanism was not defined. Thus, it is of great interest to elucidate the relationship between the post-activation trafficking regulation of STING and microtubule network, and to seek potential therapeutic intervention strategies to combat viral infections.

In this study, we report that monomethyl auristatin E (MMAE), a potent MDA commonly used in antibody-drug conjugates (ADCs) [29], enhanced STING signaling and augmented IFN and NF-kB responses. Although MMAE has been successfully used in ADC for cancer treatment, the functions of MMAE in innate immunity and its antiviral effect have not been reported so far. Detailed mechanistic studies revealed that MMAE changed the pattern of STING trafficking, increased STING-containing puncta and promoted STING oligomerization, leading to enhanced IFN productions and IFN-dependent broad-spectrum antiviral immune responses. We demonstrated that the combination of cGAMP and MMAE showed potent antiviral activity in a STING-dependent manner both *in vitro* and *in vivo*. These results provided insights into the regulatory mechanisms of microtubule structures on cGAMP-mediated STING pathway and paved the way for its potential application in antiviral immunotherapy.

## Results

### MMAE specifically enhanced cGAMP-mediated STING signaling, including the phosphorylation signaling cascades and the innate immune response

**Fig 1:**
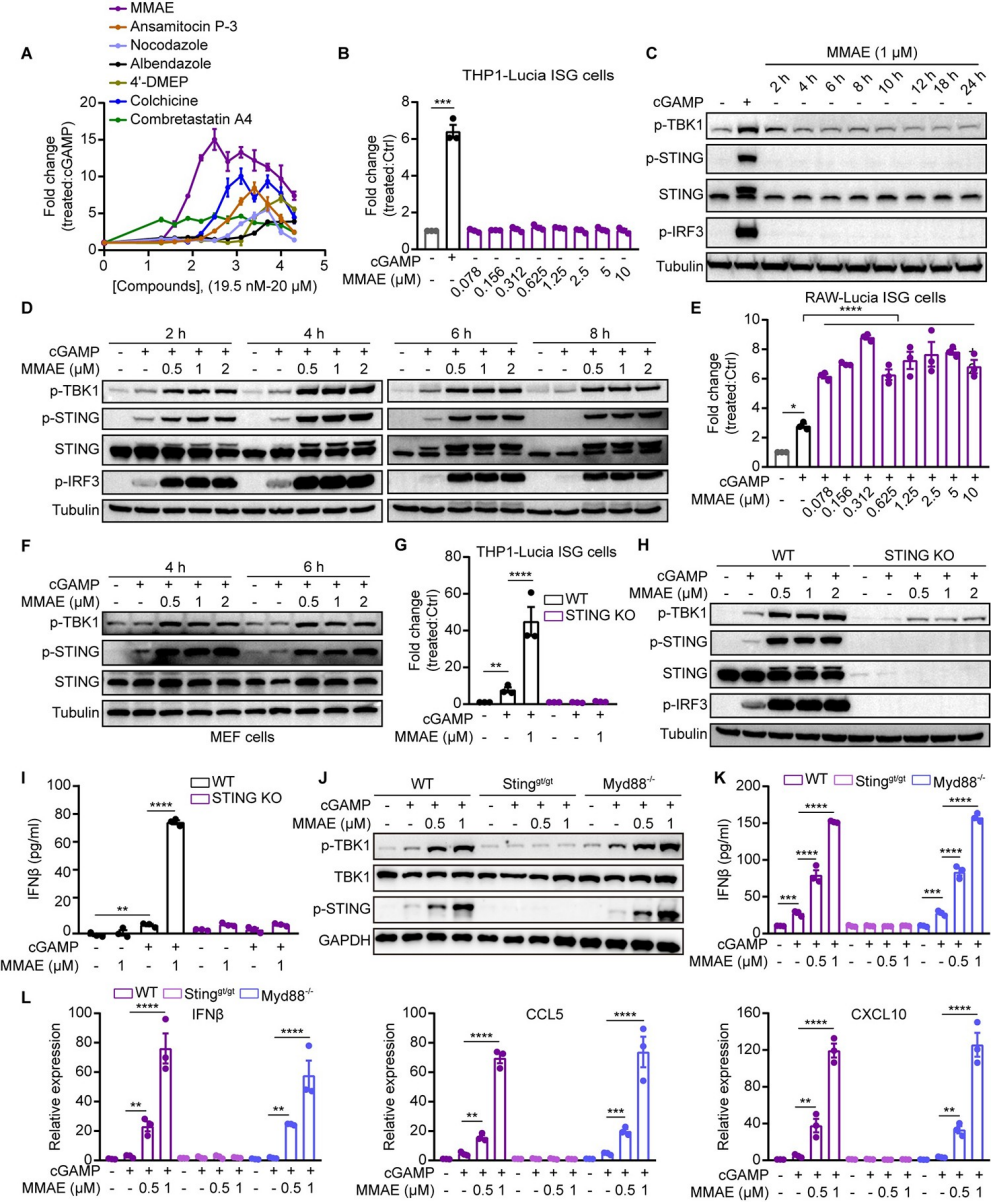
MDAs including MMAE enhanced the cGAMP-mediated STING pathway. (A, B, E, and G) THP1-Lucia ISG (WT and STING KO) and RAW-Lucia ISG cells were treated with 2’3’-cGAMP (cGAMP, 0.5 μM) and/or indicated doses of MDAs or MMAE for 24 h, and the fold change of luminescence was normalized to DMSO or cGAMP-treated cells. (C, D, F, and H) THP1-Lucia ISG (WT and STING KO) and MEF cells were treated with cGAMP (0.5 μM) and/or indicated concentrations of MMAE for indicated times (C, D and F) or 6 h (H), and activation of the STING pathway were analyzed by immunoblotting. (I-L) THP1-Lucia ISG cells (WT and STING KO) and BMDMs (WT, *Sting*^*gt/gt*^, or *Myd88*^*-/-*^ mice) were stimulated with cGAMP and/or indicated concentrations of MMAE for 12 h (I, K) or 6 h (J, L). IFNβ production was measured by ELISA analysis (I and K). the activation of STING pathway was analyzed by immunoblotting (J). mRNA expression levels of *IFNβ*, *CCL5* and *CXCL10* in BMDMs (WT, *Sting*^*gt/gt*^, or *Myd88*^*-/-*^ mice) (n = 3 biological replicates) (L). cGAMP was used at 0.5 μM for all experiments unless otherwise noted.

THP1 Lucia ISG cells express the secreted luciferase reporter gene under the control of an IRF-inducible promoter containing five IFN-stimulated response elements (ISRE), allowing the monitoring of the IRF pathway. We found that a variety of MDAs increased cGAMP-mediated luciferase signal in THP1 Lucia ISG cells through high-throughput screening (Fig 1A). To validate the findings, we investigated the effects of microtubule stabilizers (epothilone B, paclitaxel and docetaxel), DNA topoisomerase inhibitors (etoposide and topotecan), and other drugs that have microtubule-targeting activity. Indeed, only the microtubule destabilizers robustly enhanced the IRF-induced immune response in THP1 Lucia ISG cells induced by cGAMP (S1A and S1B Fig). The results suggested that the potentiation effect of MDAs on the cGAMP-mediated signaling pathway may not be through mitotic arrest and its associated cell death. To gain insights into possible mechanisms of MDAs in innate immune responses, we selected MMAE as a representative member of MDAs for further investigations because of its relatively significant potentiation effect, well-known mode of action, and microtubule disruptive activity at lower doses [29].

We first examined the effect of MMAE alone on the cGAS-STING pathway in THP1-Lucia ISG cells. Compared with cGAMP treatment, MMAE could not induce activation of the ISRE luciferase reporter (Fig 1B), nor could it induce the phosphorylation of the STING signaling cascade on its own as shown by western blot (Fig 1C). However, multiple MDAs, including MMAE, increased the activation of the cGAMP-mediated STING signaling cascade further upon cGAMP co-treatment by analyzing the phosphorylation of STING, TBK1 and IRF3 (Figs 1D, S1C and S1D). Remarkably, the potentiation effect was observed within two hours after stimulation was initiated (Fig 1D), suggesting that MMAE-mediated enhancement of STING signaling cascades is independent of mitotic arrest or cytotoxicity. We confirmed the immune potentiation effect of MMAE in RAW-Lucia ISG cells as well as in primary MEF cells (Fig 1E and 1F). Next, we investigated whether the effect of MMAE on cGAMP-mediated STING signaling was dependent on STING. THP1-Lucia ISG cells of wild type (WT) and STING-knockout (KO) were treated with cGAMP or cGAMP plus MMAE, and we found that STING KO completely eliminated cGAMP-induced ISRE reporter activity and the activation of the TBK1-STING-IRF3 signaling cascade (Fig 1G and 1H). Similarly, MMAE robustly elevated cGAMP-induced production of secreted interferon-β (IFNβ) and ISGs expression in WT but not in STING-KO THP1-Lucia ISG cells (Figs 1I and S2A).

To verify the physiological relevance of these observations, we evaluated the immune-enhancing effects of MMAE treatment in primary immune cells by using bone-marrow-derived macrophages (BMDMs) from WT, *Sting*^*gt/gt*^, and *Myd88*^*-/-*^ mice. The *Sting*^*gt/gt*^ and *Myd88*^*-/-*^ mice are specifically deficient in STING and MyD88 respectively. Similar to the results seen in THP1-Lucia ISG cells (S2B and S2C Fig), MMAE amplified cGAMP-mediated STING signaling cascade and increased the production of secreted IFNβ in WT and *Myd88*^*-/-*^ BMDMs, but not in *Sting*^*gt/gt*^ BMDMs (Fig 1J and 1K). Consistently, MMAE enhanced cGAMP-induced expression of IFNβ and ISGs in a STING-dependent manner (Fig 1L).

To determine if MMAE specifically enhanced the cGAMP-mediated STING pathway but not the RNA-sensing innate immune pathways or Toll-like receptor (TLR) signaling, THP1-Lucia ISG cells were stimulated with Sendai virus (SeV), polyinosinic-polycytidylic acid (Poly(I:C)), or lipopolysaccharide (LPS) with or without MMAE. We found that MMAE enhanced immune responses only after cGAMP treatment but not RNA- or TLR-sensing under the test conditions (S2D–S2G Fig). These results suggested that the combination of MMAE and cGAMP selectively enhanced STING signaling.

**Fig 2:**
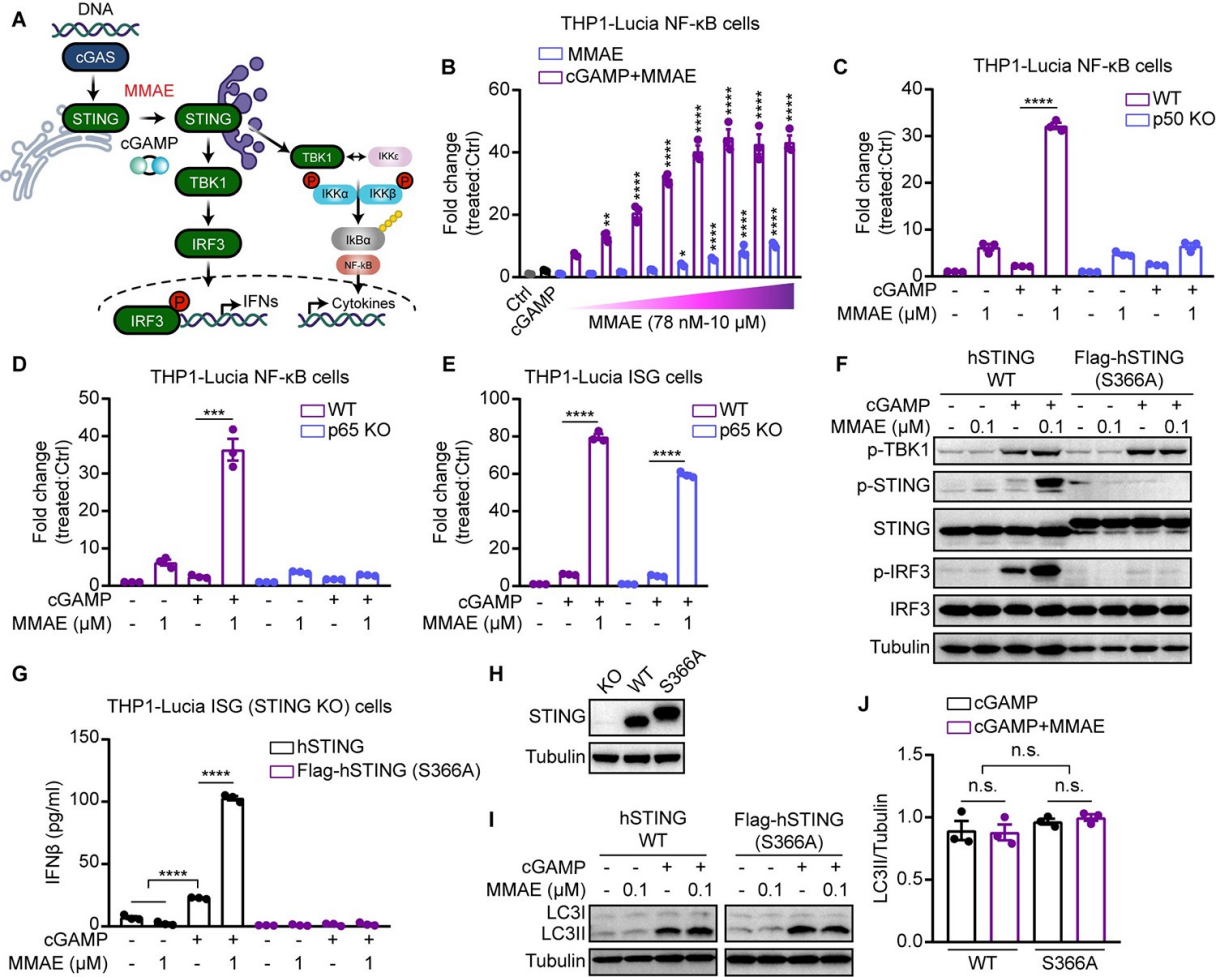
MMAE specifically enhanced the cGAMP-STING-mediated NF-kB immune response. (A) A diagram of MMAE promoting STING-mediated NF-kB signaling. (B-E) THP1-Lucia NF-kB (WT, p50 KO and p65 KO) and THP1-Lucia ISG (WT and p65 KO) were treated with cGAMP (0.5 μM) and/or indicated doses of MMAE for 24 h. The fold change of luminescence was normalized to DMSO-treated cells. (F, I and J) HEK293T cells were transiently transfected with STING plasmids (WT, Flag-hSTING (S366A)) for 24 hours. Cells were stimulated with cGAMP (0.5 μM) and/or MMAE (0.1 μM) for 6 h, and cell lysates were analyzed by immunoblotting for the indicated proteins (F and I). Quantification of LC3II/tubulin ratio from three independent experiments (J). (G) THP1-Lucai ISG (STING KO) cells stably expressing (hSTING WT, Flag-hSTING (S366A)) were stimulated with cGAMP and/or MMAE (1 μM) for 12 h. IFNβ production was measured by ELISA analysis. (H) STING was analyzed by immunoblotting in THP1-Lucai ISG (STING KO) cells.

We then tested the effect of MMAE on IFN-independent signaling of STING. Since STING could induce NF-κB signaling besides type I IFNs (Fig 2A), we tested whether the STING-mediated NF-kB immune response was enhanced by MMAE. THP1-Lucia NF-kB cells were treated with cGAMP, LPS, or IL-1β in the absence or presence of MMAE for 24 h. We found that cGAMP and MMAE alone induced weak NF-kB reporter activity (Fig 2B). Of note, we observed that MMAE specifically enhanced cGAMP-mediated STING-dependent NF-kB reporter activity, but not the NF-kB signaling induced by LPS or IL-1β (Figs 2B, S2H and S2I). Moreover, we showed that the potentiation of NF-κB signaling induced by cGAMP and MMAE co-treatment was abolished when p50 or p65 were genetically ablated in THP1-Lucia NF-kB cells (Fig 2C and 2D), whereas the potentiation of ISRE reporter activity was not affected in p65-deficient THP1-Lucia ISRE cells (Fig 2E). To characterize the effect of MMAE on STING-induced autophagy in the absence of IFN, we overexpressed STING WT and S366A mutant in HEK 293T cells (without endogenous cGAS-STING expression) and THP1-Lucia ISG (STING KO) cells, respectively. We confirmed that MMAE enhanced cGAMP-mediated STING-IRF3 phosphorylation cascade and IFNβ production only in cells with WT STING expression, but not in cells with STING S366A mutant (Fig 2F–2H). This is consistent with the reports that the STING S366A mutant impaired the phosphorylation of STING and IRF3 and downstream signaling. Moreover, we showed that both the WT and S365A mutant of STING could activate autophagy in response to cGAMP. Of note, MMAE did not strongly enhance cGAMP-STING-mediated autophagy (Fig 2I and 2J). Together, our results suggested that MMAE robustly and specifically enhanced the cGAMP-STING-mediated type I IFNs and NF-kB signaling in tested cells.

### MMAE boosted immune responses induced by distinct CDNs via the STING-IRF3 pathway directly

**Fig 3:**
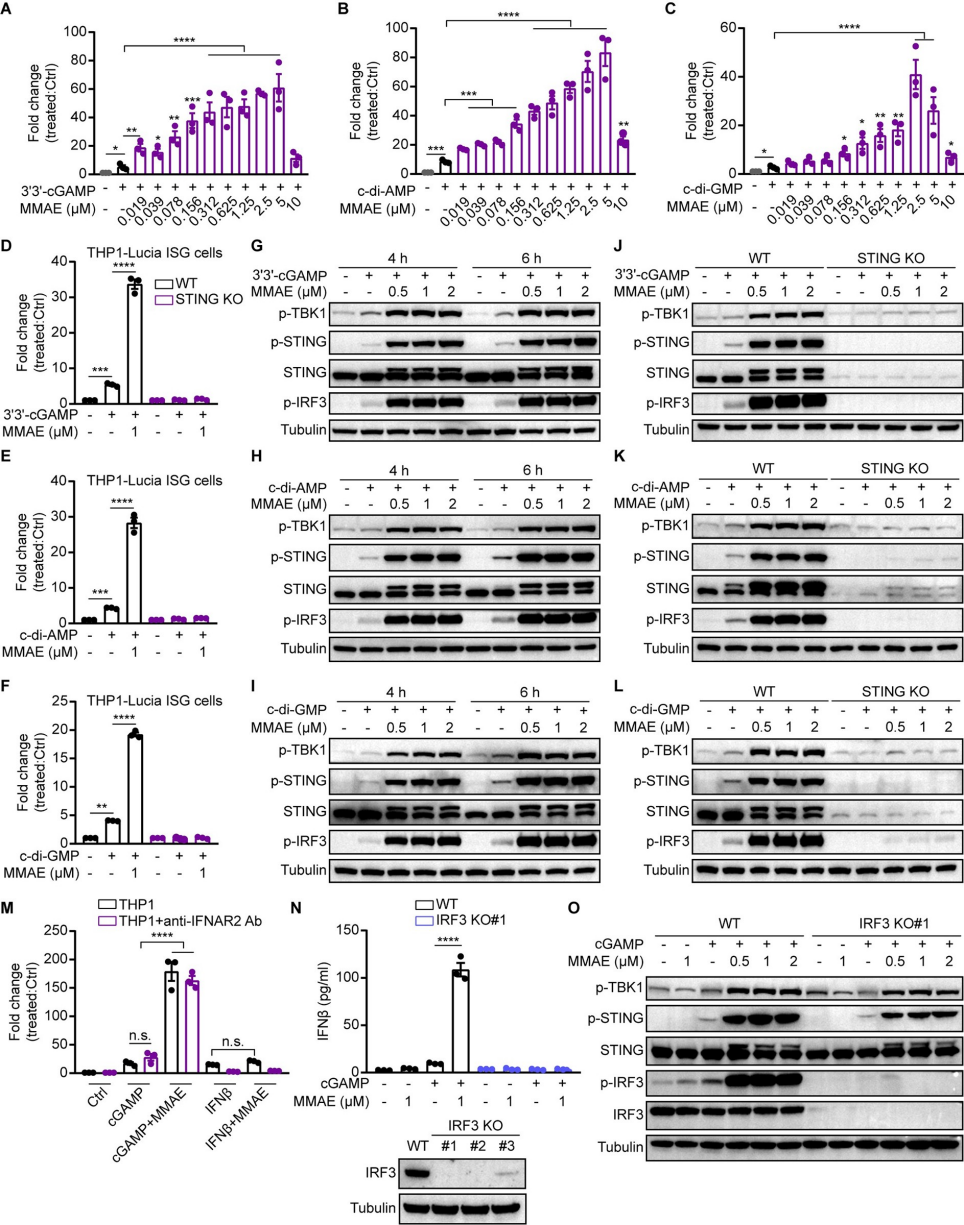
MMAE enhanced the activation of STING pathways mediated by distinct CDNs in a direct STING-IRF3 dependent manner. (A-L) THP1-Lucia ISG (WT and STING KO) cells were treated with cGAMP with or without indicated doses of MMAE for 24 h (A-F) or 6 h (J-L), and the fold change of luminescence was normalized to DMSO-treated cells. The activation of STING pathway was analyzed by immunoblotting (G-L). (M) THP1-Lucia ISG cells were pretreated for 12 h with or without anti-IFNAR2 antibody (20 μg/ml), and then stimulated with cGAMP or IFNβ (200 pg/ml) for 24 h in the absence or presence of MMAE (1 μM). Fold change of luminescence was normalized to DMSO-treated cells. (N and O) THP1-Lucia ISG (WT and IRF3 KO) cells were treated with cGAMP and/or indicated doses of MMAE for 12 h (N) or 6 h (O). IFNβ production was measured by ELISA analysis (N). Expression of IRF3 and the activation of STING pathway was analyzed by immunoblotting (N and O).

To further define the relevance of MMAE-enhanced cGAMP-STING pathway, we treated THP1-Lucia ISG cells with bacterial-derived CDNs, including 3’3’-cGAMP, c-di-AMP, and c-di-GMP. As expected, MMAE robustly enhanced the immune responses induced by bacterial-derived CDNs in WT THP1 reporter cells, but this effect was completely abolished in STING-KO cells (Fig 3A–3F). Consistently, the phosphorylation cascade of the STING signaling pathway induced by bacterial-derived CDNs was boosted upon MMAE treatment in WT (Fig 3G–3I) but not STING-KO THP1-Lucia ISG cells (Fig 3J–3L). These results suggested that MMAE had a ubiquitous potentiation effect on STING activation induced by distinct CDNs.

Since both cGAMP and human IFNβ could induce ISRE reporter activity, to further understand whether MMAE enhanced the cGAMP-mediated induction of ISGs through the canonical STING-IRF3 signaling pathway directly or the interferon-α/β receptor (IFNAR) signaling pathway in an indirect manner (S3A Fig), we took both genetic and pharmacological approaches. We found that IFNβ-induced ISRE reporter activity was abolished by anti-IFNα/β receptor 2 (IFNAR2) antibody, whereas cGAMP-induced immune activity was not affected by IFNAR2 antibody (Fig 3M). By contrast, the ISRE immune response induced by cGAMP was significantly enhanced by MMAE, whereas IFNβ-induced immune activity was not promoted by MMAE (Fig 3M). Moreover, the enhancement of IFNβ production and IRF3 phosphorylation mediated by combination of cGAMP and MMAE were eliminated when IRF3 was genetically ablated (Fig 3N and 3O). These results indicated that MMAE enhanced cGAMP-induced ISRE immune activity mainly through the TBK1-STING-IRF3 signaling pathway directly. In addition, we stimulated THP1-Lucia ISG cells with an IFNAR2 agonist, RO8191, which directly binds to IFNAR2 and activates the expression of ISGs. We found that RO8191 activate ISRE luciferase reporter, while MMAE could only slightly enhance the effects of RO8191 (S3B Fig). Furthermore, cGAMP-mediated phosphorylation of the STING signaling cascade was enhanced by MMAE, but not activated by the combination of RO8191 and MMAE (S3C Fig). STAT1, STAT2, or STAT3 KO had no effect on the potentiation effect of cGAMP plus MMAE (S3D–S3I Fig). Together, these results confirmed that MMAE boosted immune responses directly via the cGAMP-mediated STING-IRF3 signaling pathway in our tested cellular conditions.

### MMAE promoted the cGAMP-STING pathway by increasing puncta number and the extent of STING oligomerization

**Fig 4:**
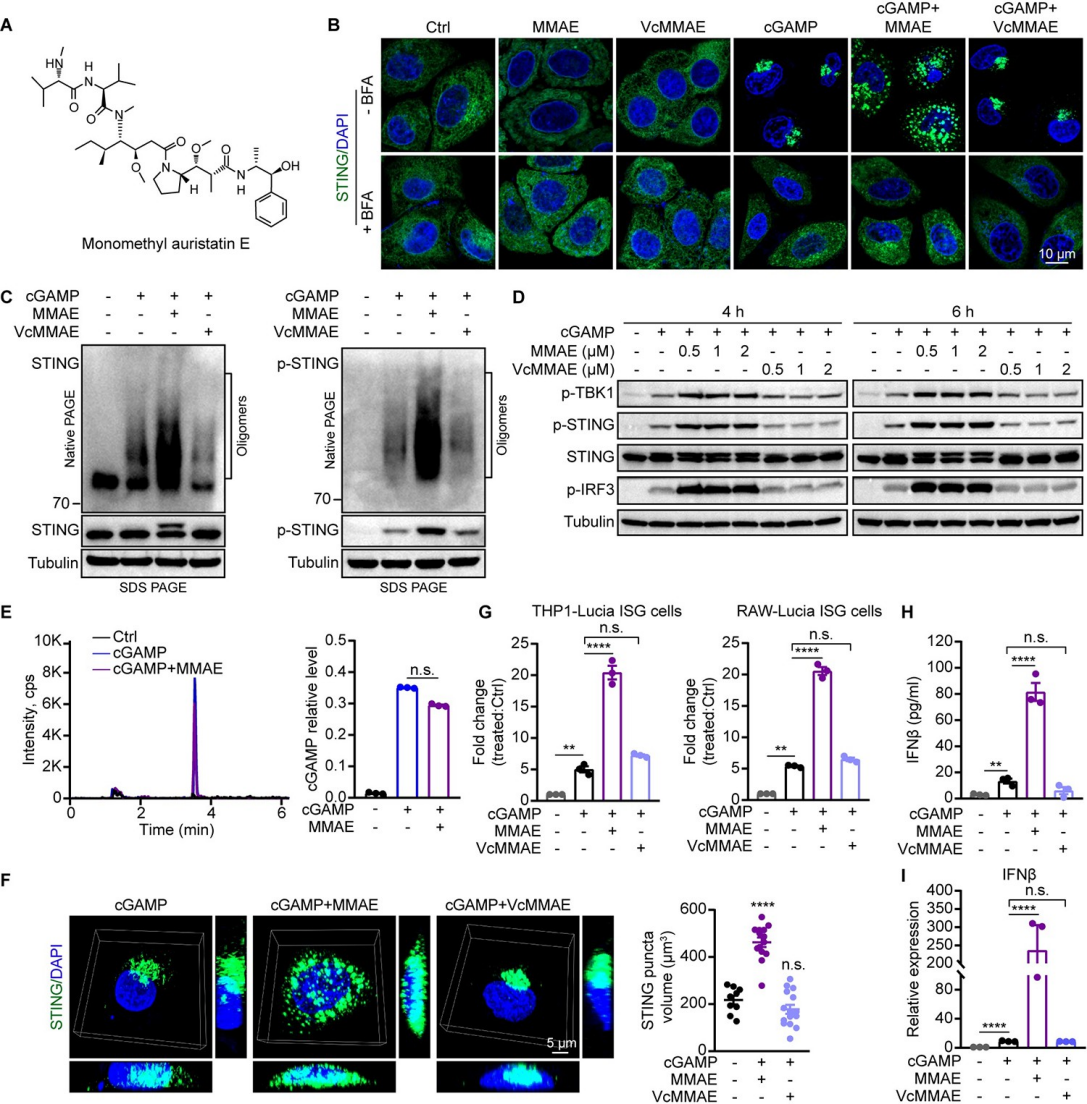
MMAE boosted the cGAMP-mediated STING pathway by increasing STING-containing membrane puncta numbers and the extent of STING oligomerization. (A) Chemical structure of Monomethyl auristatin E (MMAE). (B) HeLa cells stably expressing human STING-GFP were stimulated with cGAMP (8 μM) and/or MMAE (1 μM), or VcMMAE (1 μM) for 2 h in the absence or presence of brefeldin A (BFA, 1 μM). Fluorescent images of cells were acquired on a Zeiss LSM980 Airyscan2 Confocal microscope using a 63× (NA 1.45) objective and processed in Zen Blue 3.1 software. STING (green), nuclei were stained with Hoechst (blue). Scale bars, 10 μm. (C-E) THP1-Lucia ISG cells were stimulated with cGAMP with or without MMAE (0.5 μM) or VcMMAE (0.5 μM) for 4 h (C and E). STING oligomerization was analyzed by native PAGE, and indicated proteins were detected by immunoblotting. The results are representative of three independent biological replicates (C). The activation of STING pathway was analyzed by immunoblotting (D). cGAMP quantification by LC-MS/MS in THP1-Lucia ISG cell lysates (E). (F) HeLa cells are stimulated similarly as in B. The STING (green) puncta are shown as 3D projections of Z-stack images. Scale bar, 5 μm. The STING puncta volume was quantitated by Imaris software (version 9.7) (n = 20). (G) THP1-Lucia ISG and RAW-Lucia ISG cells were treated with cGAMP with or without MMAE (1 μM), or VcMMAE (1 μM) for 24 h. Fold change of luminescence was normalized to DMSO-treated cells. (H and I) THP1-Lucia ISG cells were stimulated by cGAMP with or without MMAE (1 μM), or VcMMAE (1 μM) for 12 h (H), or 6 h (I). IFNβ induction was measured by ELISA and qPCR analysis.

The CDNs trigger the translocation of STING from ER to perinuclear region, where it forms puncta-like structures and activates TBK1 and IRF3 to induce the production of IFNs and other immune-modulating molecules. To explore the dynamic regulatory mechanism of MMAE on cGAMP-mediated STING activation, we constructed HeLa cells stably expressing hSTING-GFP. There were no differences in the distribution pattern of hSTING-GFP between untreated cells and cells treated with MMAE alone (Fig 4A and 4B). Consistent with our previous results, cGAMP significantly induced STING translocation and perinuclear puncta formation [5] (Fig 4B). By contrast, co-treatment of the cells with MMAE and cGAMP caused STING perinuclear puncta to disperse into numerous small vesicles throughout cytoplasm (Fig 4B). VcMMAE [30], which was employed as a negative control, failed to modulate cGAMP-mediated STING trafficking and translocation (Figs 4B and S4A). Moreover, brefeldin A, which is a specific inhibitor of protein trafficking by blocking the transport of membrane proteins from ER to Golgi apparatus [21], effectively inhibited STING translocation (Fig 4B). To verify the distinct STING trafficking patterns mediated by cGAMP or MMAE plus cGAMP, we treated HeLa cells (hSTING-GFP) with other CDNs, such as 3’3’-cGAMP and c-di-AMP. Similarly, MMAE caused dispersion of the STING perinuclear puncta induced by 3’3’-cGAMP or c-di-AMP (S4B Fig). Consitenty, STING perinuclear puncta induced by herring testes DNA (HT-DNA, a double strand DNA sensed by cGAS to produce cGAMP) can also be scattered by MMAE (S4B Fig).

To confirm that STING signaling was maintained or even enhanced in the dispersed vesicles of STING, we extracted membrane fractions containing STING (supernatant, S1) from cell homogenate after centrifugation. We detected the oligomerization and activation of STING by native PAGE, confirming that MMAE induced more cGAMP-dependent STING oligomers than cGAMP alone or cGAMP plus VcMMAE (Fig 4C). Accordingly, phosphorylated STING signaling was significantly enhanced by MMAE (Fig 4C and 4D). To rule out the possibility that MMAE amplifies STING immune responses by promoting cGAMP entering into cells, we quantified intracellular cGAMP concentrations by liquid chromatography-tandem mass spectrometry. There were no significant differences in intracellular cGAMP levels after cGAMP treatment in the absence or presence of MMAE (Fig 4E). The results demonstrated that MMAE promoted STING oligomerization and vesicle dispersion which led to enhanced STING signaling.

We then assessed the number of STING puncta formed upon treatment with cGAMP with or without MMAE or VcMMAE. MMAE, but not VcMMAE, significantly increased the number of STING puncta induced by cGAMP treatment (Fig 4F). The positive effect of MMAE and the negative effect of VcMMAE were confirmed by the luciferase reporter assay with THP1/RAW-Lucia ISG cells (Figs 4G and S4C). Similarly, MMAE, but not VcMMAE, significantly promoted cGAMP-mediated production of secreted IFNβ and ISGs expression (Figs 4H, 4I and S4D). Collectively, these data strongly suggested that MMAE promoted the cGAMP-mediated STING pathway by increasing puncta number and the extent of STING oligomerization.

### MMAE altered the trafficking pattern of STING and delayed STING degradation

**Fig 5:**
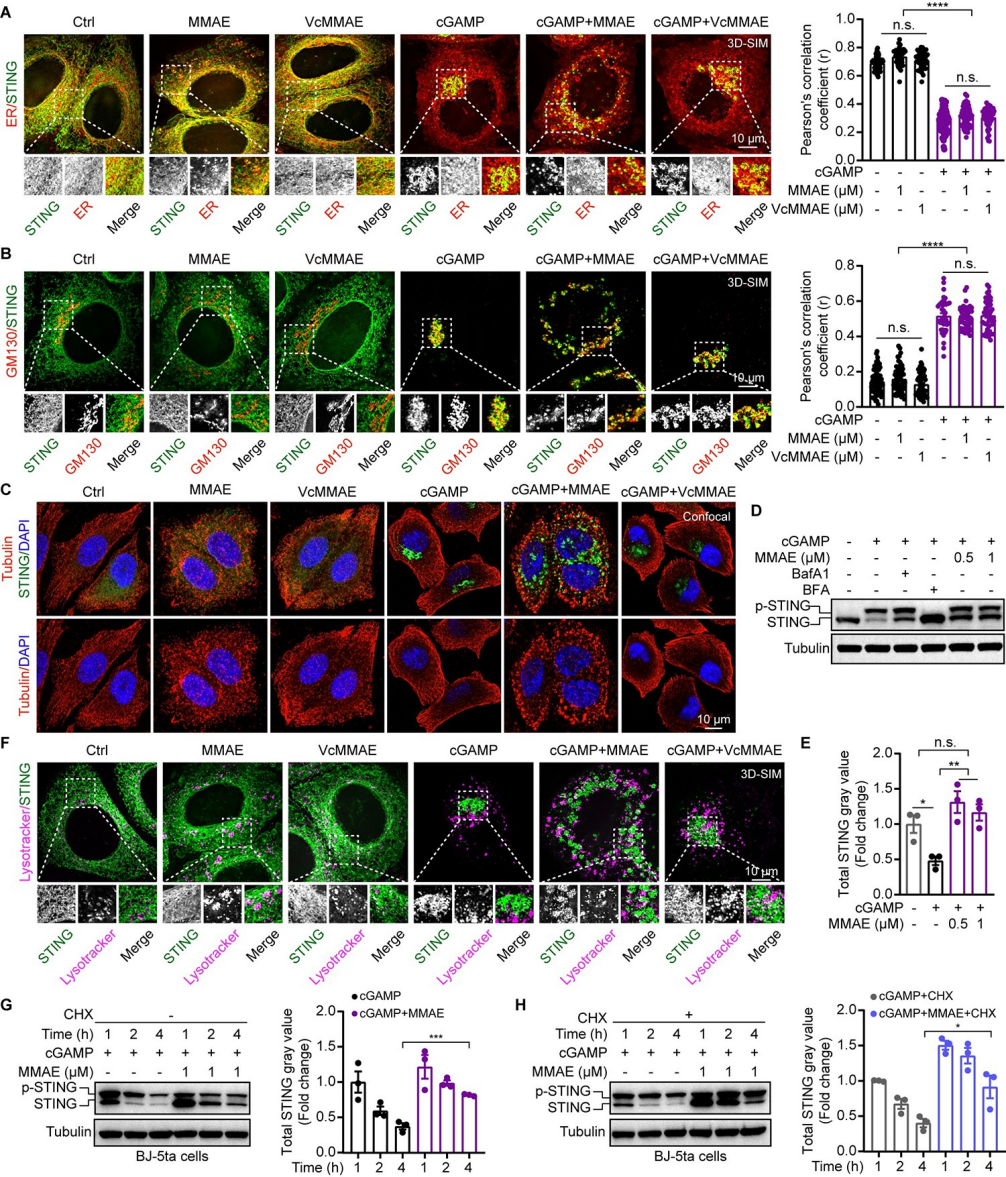
MMAE changed STING trafficking routes and promoted cGAMP-mediated STING activity. (A-C and F) HeLa cells stably expressing human STING-GFP were stimulated with cGAMP (8 μM) and/or MMAE (1 μM), or VcMMAE (1 μM) for 2 h. (A and F) Live cells were stained by ER-Tracker Blue-White DPX and LysoTracker Deep Red. (B and C) Cells were fixed, permeabilized, and stained for GM130 (a Golgi protein, red) or tubulin (red). Nuclei were stained with DAPI (blue). All structured illumination microscope (3D-SIM) images are z-stack images. Scale bars, 10 μm. 3D-SIM images was acquired and processed using the Highly Intelligent and Sensitive SIM (HIS-SIM), and Wiener deconvolution was used in reconstructed images. Dashed white boxes in each main image indicate enlarged areas of interest shown below. Co-localization was quantified using Pearson’s correlation coefficient (r), shown on the right of each row of images (n = 50). (D and E) BJ-5ta cells were stimulated with cGAMP (8 μM) with or without MMAE (pre-treatment for 30 min), bafilomycin A1 (BafA1, 100 nM), or brefeldin A (BFA, 1 μM) for 2 h. Total STING protein was quantified by image J software (n = 3 biological replicates). (G and H) STING stability was analyzed by immunoblotting in the absence or presence of cycloheximide (CHX, 50 μg/ml). BJ-5ta cells were treated and analyzed as in (D and E).

To further explore the mechanism for STING dispersal at an accurate spatial and temporal resolution, we performed live-cell time-lapse imaging and ER-Tracker live-cell staining using HeLa cells stably expressing hSTING-GFP (Figs 5A and S5A and S1 Movie). In line with the results in Fig 4B, we found that cGAMP induction caused strong activation of STING trafficking, moving from ER to perinuclear region within 60 minutes (Figs 5A and S5A and S1 Movie). Interestingly, MMAE treatment changed the cGAMP-mediated STING trafficking routes, resulting in STING vesicles to move aimlessly and scatter throughout the cytoplasm (S1 Movie and S5A Fig). STING is a transmembrane protein on ER and its activities are dynamically regulated by vesicular trafficking. Previous studies have shown that proper distribution and movement of vesicles and organelles within the cytoplasm is highly dependent on the rapid assembly and disassembly of microtubules [31]. We suspect that MMAE might disrupt cGAMP-mediated STING trafficking from ER to Golgi apparatus along microtubules by inhibiting tubulin polymerization. Imaging data revealed that MMAE, as an MDA, disrupted microtubule network and caused Golgi apparatus dispersal [29,32], whereas VcMMAE treatment at the same concentration had no such an effect (Fig 5B and 5C). Similarly, the disruption of microtubule network and STING dispersal phenotype were also confirmed by other MDAs (microtubule stabilizer paclitaxel is used as a negative control) (S5B Fig). Notably, although MMAE changed distribution pattern of STING and Golgi in the MMAE plus cGAMP group, STING is still colocalized with Golgi (Fig 5B). Together, these data indicated that MDAs dispersed the Golgi apparatus and altered STING trafficking routes by disrupting the microtubule network.

Endogenous STING protein is quickly degraded by lysosome after stimulation by CDNs. Prevention of STING degradation could potentially lead to sustained STING signaling and immune responses, providing therapeutic advantages in antiviral treatment. To characterize the STING degradation, we employed BJ-5ta and THP1 cells, which are two cell lines with robust STING-degrading signaling upon cGAMP stimulation. To investigate the effect of MMAE on STING degradation, we stimulated BJ-5ta and THP1 cells with a high concentration of cGAMP with or without MMAE and assessed STING degradation by measuring STING protein amounts. Consistent with previous studies, bafilomycin A1 (BafA1, inhibitor of lysosome function) or brefeldin A treatment potently blocked STING degradation. We found that the amounts of total STING were accumulated after MMAE plus cGAMP co-treatment compared to cGAMP treatment alone (Fig 5D and 5E), indicating that co-treatment delayed STING degradation. Furthermore, when protein synthesis is stopped by cycloheximide, MMAE significantly inhibited the degradation of STING over time in BJ-5ta and THP1 cells (Figs 5G, 5H and S5C). STING puncta were surrounded and engulfed by lysosome after cGAMP treatment. However, both lysosome and STING were dispersed with the addition of MMAE (Fig 5F), which might cause delayed degradation of STING. Together, these data provide evidence that MMAE not only disrupted microtubule structure and altered STING trafficking, but also delayed STING degradation, leading to a stronger and more sustained cGAMP-STING signaling cascade.

### MMAE significantly promoted cGAMP-mediated STING antiviral immunity in the cell

**Fig 6:**
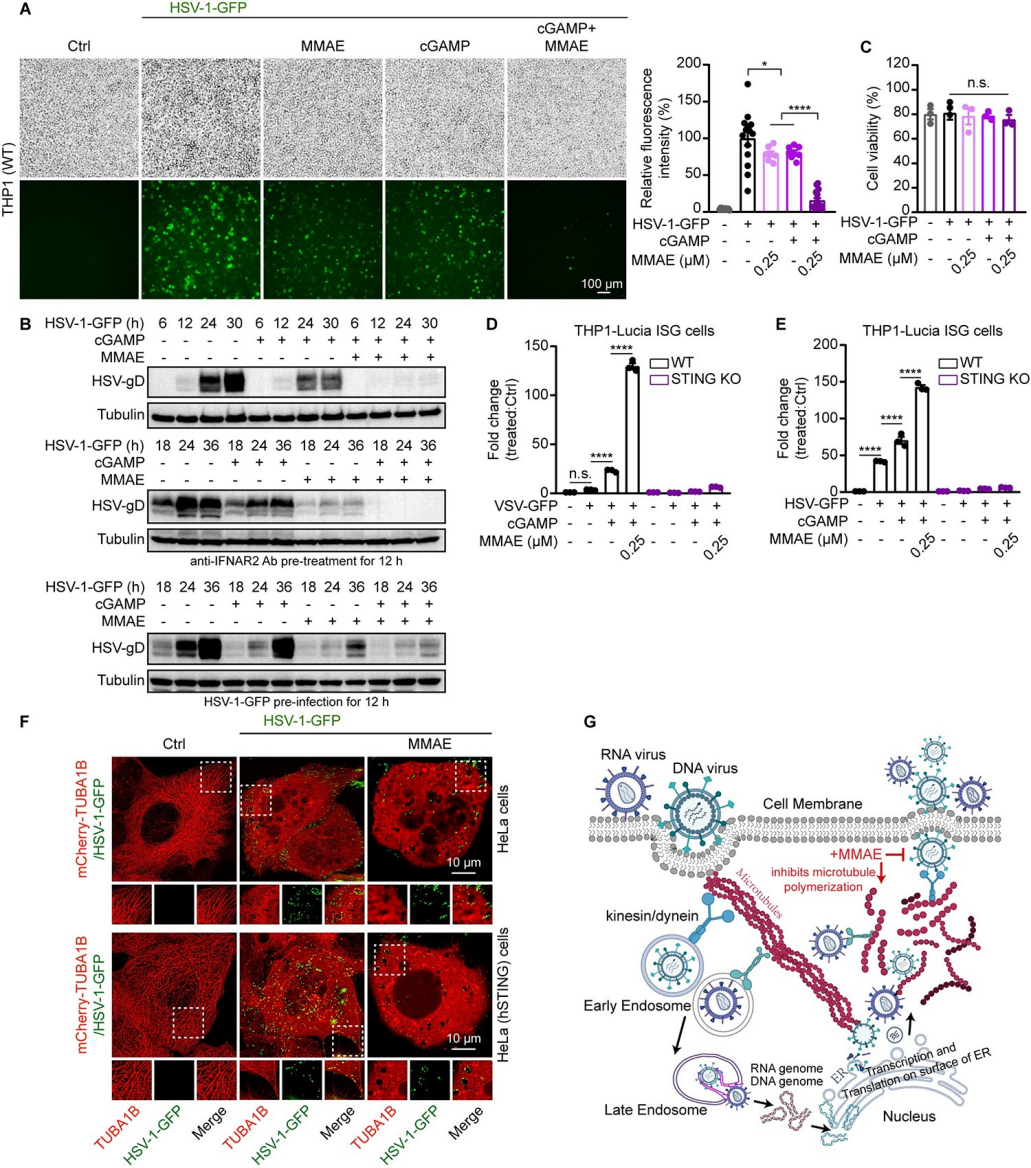
MMAE alone had antiviral effects and also further amplified the cGAMP-mediated antiviral immune response. (A and C-E) THP1-Lucia ISG cells (WT and STING KO) were infected with HSV-1-GFP (MOI = 1) or VSV-GFP (MOI = 0.1), and then cultured with cGAMP and/or MMAE (0.25 μM) for 24 h. The cells were then imaged with Olympus IX83 Inverted fluorescence microscope (A). The fluorescence intensity of virus-GFP was determined by ImageJ software, shown on the right of each row of images (n = 15, biological replicates). Scale bars, 100 μm. Cell viability was determined by ATP assay after indicated treatments (C). ISRE reporter activity was measured, and the fold change of luminescence was normalized to DMSO-treated cells (D and E). Bars are the means ± SEM. Significance was determined by one-way ANOVA; *p < 0.05, **p < 0.01, ***p < 0.001, ****p < 0.0001, n.s. means non-significant. (B) THP1-Lucia ISG cells were infected with HSV-1-GFP (MOI = 1), and then cultured with cGAMP and/or MMAE (0.25 μM) with or without pretreatment of HSV-1-GFP (MOI = 1) or anti-IFNAR2 antibody (20 μg/ml) for indicated times. Expression of viral gene was determined by immunoblotting. (F) HeLa (STING deficient) and HeLa hSTING cells stably expressing human mCherry-TUBA1B were infected with HSV-1-GFP (MOI = 1), and then cultured with or without MMAE (0.1 μM) for 18–24 h. Representative confocal images of virus transport along microtubules were shown. Enlarged insets highlighted the co-localization of the viral particles (dashed white boxes) with intracellular microtubules. mCherry-TUBA1B (red), HSV-1-GFP (green). Scale bars, 10 μm. (G) Schematic diagram of microtubule-based transport of virus entry, replication, assembly, and egress from the host cell. MMAE-mediated microtubule network disruption seriously affects every process of viral replication and reproduction.

Activation of STING induces the production of IFNs and plays a critical role in controlling viral infections. Using herpes simplex virus 1 (HSV-1) and vesicular stomatitis virus (VSV) as an example of DNA and RNA virus, respectively, we examined the antiviral activity of MMAE and cGAMP combination. THP1 and L929 cells were infected with GFP-tagged herpes simplex virus 1 (HSV-1-GFP) or vesicular stomatitis virus (VSV-GFP) for 24 h. The mean fluorescence intensity (MFI) of viral GFP was used as an indicator of viral propagation. Both cGAMP [33,34] and MMAE on its own inhibited viral propagation (Figs 6A, S6A and S6C), consistent with previous reports on antiviral activities of MDAs [27,35]. Microtubule depolymerization not only disrupts virus trafficking in the endosome, but also affects the escape of virus particles from the endosome [36,37]. Of note, MMAE in combination with cGAMP almost completely inhibited viral propagation in the cell (Figs 6A, S6A and S6C). When STING was genetically ablated, the cGAMP-mediated antiviral effect was abolished. By contrast, the antiviral effect of MMAE alone was not affected by STING-KO (S6B, S6D–S6F Fig). The antiviral ability of MMAE plus cGAMP in STING-deficient cells became the same as that of MMAE alone, indicating that the enhanced antiviral effect of MMAE plus cGAMP is dependent on the STING signaling pathway (S6B and S6E Fig). Immunoblotting analysis further confirmed that MMAE and cGAMP together significantly inhibited viral reproduction in the cells over time (Figs 6B and S7A). The viral infection still could be inhibited even the co-treatment was added after HSV-1-GFP virus infection or anti-IFNAR2 antibody pretreatment (Fig 6B). Meanwhile, the effect of treatment on cell viability could be ignored under the test conditions (Figs 6C and S7B). To explore the antiviral spectrum of MMAE plus cGAMP, we infected THP1 and L929 cells with pseudorabies virus (PRV-GFP, a DNA virus), vaccinia virus (VACV-GFP, a DNA virus), and enterovirus 71 (EV-A71, an RNA virus). We found that MMAE and cGAMP in combination provided cells with significant protection against all three tested viruses (S7C and S7D Fig). We further confirmed that MMAE could enhance cGAMP-mediated STING signaling during infections by DNA or RNA viruses (Fig 6D and 6E). Together, these results suggested that the combination of cGAMP and MMAE had potent synergistic antiviral activity in a STING-dependent manner and could be used as a broad-spectrum antiviral strategy.

To see whether viral activity is modulated by MMAE-mediated microtubule destabilization, we constructed HeLa cells (STING deficient) stably expressing mCherry-TUBA1B. Imaging data revealed that MMAE at low doses dramatically disrupted microtubule network (Fig 6F). Importantly, in cells infected with HSV-1-GFP, HSV-1-GFP is localized along the microtubule, and MMAE significantly changed HSV-1-GFP invasion routes. HSV-1-GFP is trapped at certain positions inside the cells with MMAE treatment, regardless of STING expression level (Fig 6F and 6G). This is consistent with large number of reports on the role of microtubules in viral cycles. The intracellular transport of viral particles in the host cells, including particle trafficking at later stages of viral life cycles, is heavily dependent on the integrity of the microtubule network [38]. Collectively, our findings reveal a novel role of microtubule destabilizer MMAE, and its combination with cGAMP provides a promising therapeutic approach for future antiviral therapy.

### MMAE promoted the antiviral effect of cGAMP *in vivo* in a STING-dependent manner

**Fig 7:**
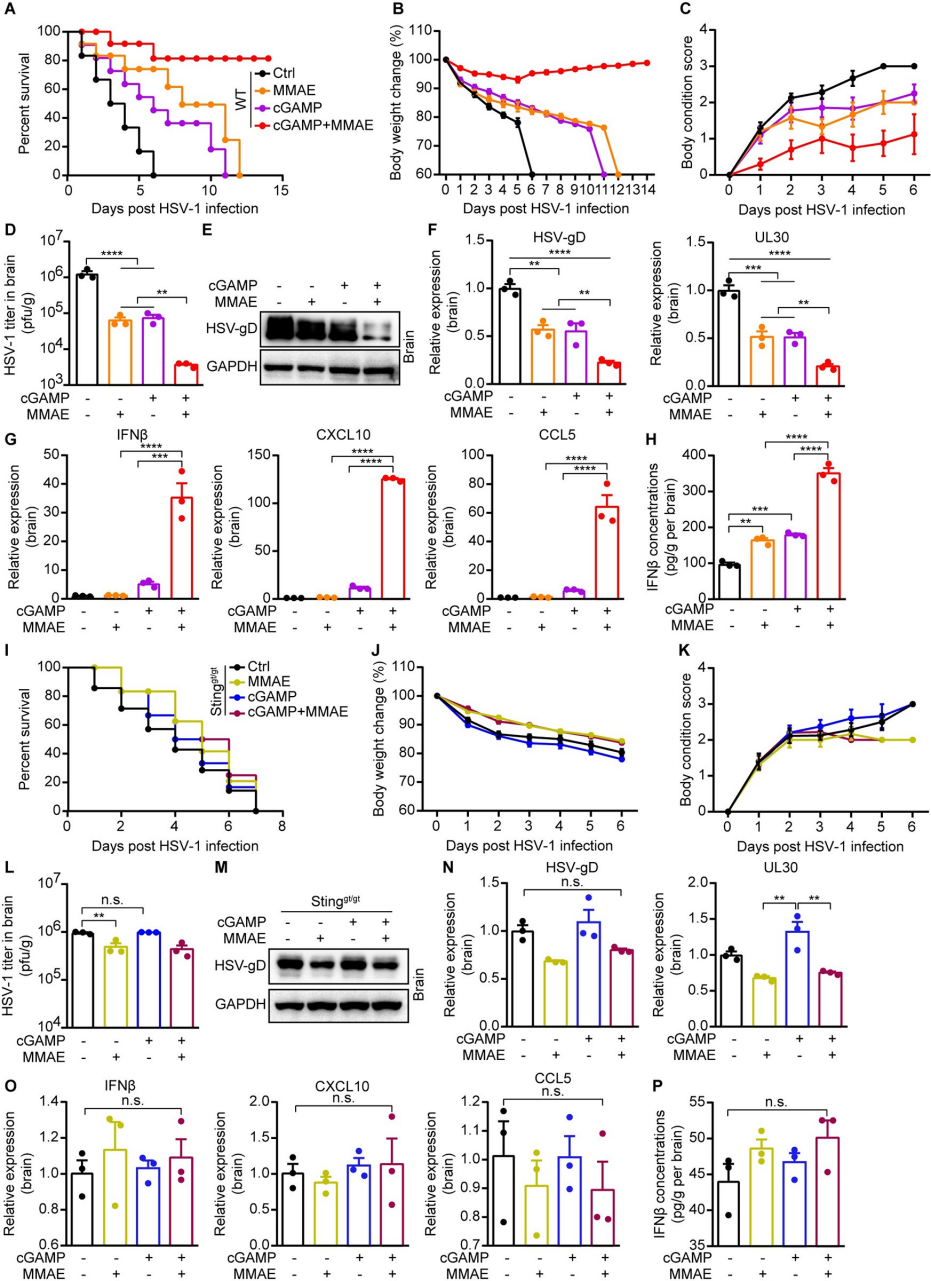
MMAE relied on the host STING pathway to enhance cGAMP antiviral immune response. (A-H and I-P) WT and *Sting*^*gt/gt*^ C57BL/6 mice (n = 10) were treated with PBS, cGAMP (30 μg/mice), MMAE (0.5 mg/kg), or cGAMP along with MMAE by intraperitoneal injection (i.p.) for 2 h. Then, the mice were infected intravenously with HSV-1-GFP at 2 × 10^8^ pfu per WT mouse or at 1 × 10^7^ pfu per *Sting*^*gt/gt*^ mouse. (A and I) Survival curves of virus-infected mice after treatments were analyzed using the log-rank (Mantel-Cox) test. (B, C and J, K) Body weight and body condition score of mice were observed and recorded daily. Body condition score was measured and calculated as in previous research with minor modifications [52] (normal = 0). (D-H and L-P) Six days after virus infection, three C57BL/6 mice (WT and *Sting*^*gt/gt*^) were randomly selected for subsequent experiments. (D and L) The viral titers in mouse brains were measured by qRT-PCR assay (n = 3 biological replicates). (E, F, M, and N) Expressions of viral genes in brains were measured by immunoblotting and qPCR analysis (n = 3). (G and O) Expressions of IFNβ and ISGs in brains were analyzed by qPCR analysis (n = 3). (H and P) IFNβ production in brains were qualified by ELISA assay.

The cGAS-STING pathway plays pivotal roles in controlling HSV-1 propagation in mice. To investigate the antiviral effect of MMAE *in vivo*, we treated HSV-1-GFP infected WT and *Sting*^*gt/gt*^ mice with cGAMP, MMAE, or in combination. We found that HSV-1-GFP infected mice treated with cGAMP or MMAE alone showed higher survival rates compared with the control group, while the mice group treated with MMAE and cGAMP together showed the highest survival rate (Fig 7A). It’s worth noting that HSV-1-GFP infected mice experienced dramatic body weight loss before death, treatment with cGAMP or MMAE alone alleviated this weight loss symptom, and the cGAMP and MMAE combination largely prevented body weight loss and body condition score decrease caused by viral infection (Figs 7B, 7C and S8A–S8C). Consistently, viral titers and the expressions of viral genes were reduced in drug treated mice, especially in the MMAE plus cGAMP group (Figs 7D–7F and S8D–S8F). Crucially, MMAE robustly promoted cGAMP-induced IFNβ production and the expression of ISGs in WT mice compared with cGAMP and MMAE alone (Figs 7G, 7H and S8G), but not in *Sting*^*gt/gt*^ mice (Figs 7O, 7P and S8H). Consistent with this data, we found that *Sting*^*gt/gt*^ mice were more vulnerable to HSV-1 infection, resulting in death even with a much lower virus titer than WT mice. In addition, MMAE alone did not prevent the death of *Sting*^*gt/gt*^ mice caused by HSV-1 infection (Fig 7I). It did not alleviate body weight loss and body condition score worsening in these mice, and viral titers or the expressions of viral genes in brain and spleen were not significantly changed, indicating a slight or undetectable antiviral effect of MMAE in *Sting*^*gt/gt*^ mice (Figs 7J–7N, S8I and S8J).

We then tested whether the combination strategy of MMAE and cGAMP could be employed to treat infection *in vivo* after virus replication had been well established. We infected mice with HSV-1-GFP first and treated the mice with MMAE, cGAMP, or in combination 16 hours later after the viral infection. We further confirmed that the combination of cGAMP and MMAE substantially decreased viral titers and the expressions of viral genes as compared with monotherapy (S8K–S8P Fig). These results suggested that the MMAE and cGAMP co-treatment strategy still could be used after the viral infection. Together, we demonstrated that MMAE promoted cGAMP-mediated host antiviral immunity in a STING-dependent manner and protected mice form HSV-1 infection *in vivo*. These data suggest that the combination of MMAE and cGAMP has great potential in antiviral immunotherapy.

## Discussion

In this study, by using MMAE as an example we show that MDAs could act synergistically with cGAMP to defend viral infections by modulating STING signaling and increasing STING-mediated immune response. We showed here that the combination of MMAE with cGAMP displayed potent and broad-spectrum antiviral activity in the cell and provided protection against HSV-1 infection in a mouse model.

**Fig 8:**
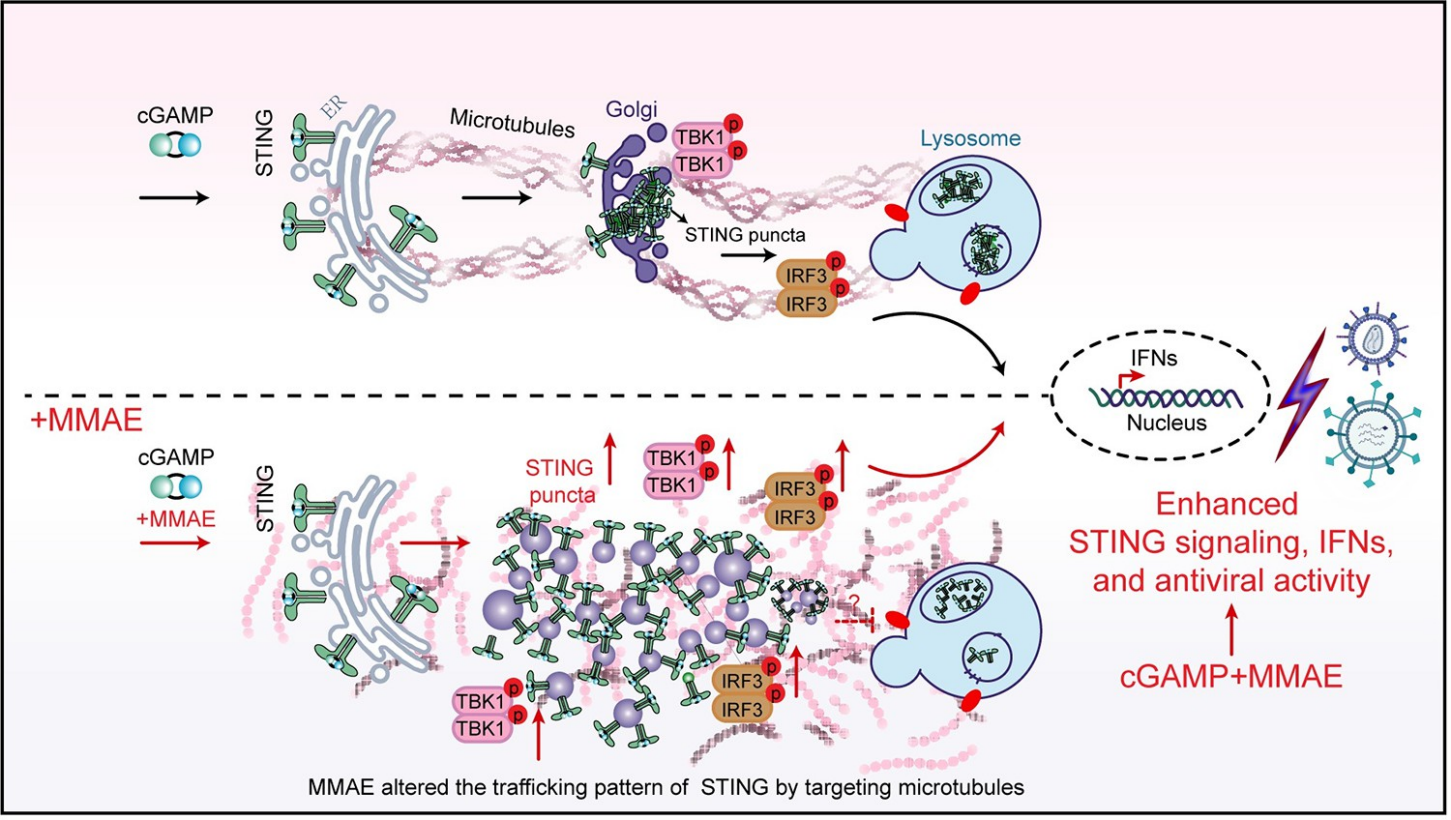
A graphic model for MMAE enhanced cGAMP-mediated antiviral immunity. MMAE changed cGAMP-mediated STING trafficking routes from ER to Golgi apparatus by disrupting the microtubule network, and delayed the trafficking-mediated STING degradation. MMAE dispersed the cGAMP-mediated STING perinuclear puncta into large number of tiny vesicles throughout the cytoplasm. The accumulated STING vesicles further amplified the cGAMP-mediated TBK1-STING-IRF3 signaling cascade, and promoted the production of IFNs and ISGs expression. MMAE alone restricted viral replication and infection by destroying microtubule networks, while MMAE combined with cGAMP exerted potent and broad-spectrum antiviral activity *in vitro* and *in vivo* in a STING-dependent manner.

Recent studies have demonstrated that STING agonists evoke potent innate immune responses via the induction of type I IFNs and IFN-stimulated genes. cGAMP, the second messenger of the cGAS-STING pathway, is critical for this host innate immune responses against viral infections. Accordingly, some viruses could degrade 2’3’-cGAMP using specific enzymes to restrict cGAMP-STING signaling [39,40], while some could cleave or repress cGAS to decrease cGAMP production [41–44]. Therefore, the strategies to enhance cGAMP (or cGAMP analog)-induced STING innate immune response may have potentials in antiviral treatment. In this study, we found that the combination of MMAE and cGAMP might be able to fulfill this purpose. We showed that MMAE enhanced cGAMP-mediated antiviral immune response by promoting the oligomerization of STING and increasing STING puncta. The imaging data clearly showed that MMAE treatment disrupted the microtubule network, dispersed the Golgi apparatus, and completely disturbed the normal transport routes of intracellular STING vesicles (Fig 8). Notably, MMAE delayed trafficking-mediated STING degradation by altering lysosome localization patterns. These results suggested that MMAE could lead to sustained and reinforced STING-dependent immune response, culminating in the production and secretion of antiviral cytokines. Moreover, our results suggested that MMAE directly regulated cGAMP- STING signaling, but not the secondary interferon-α/β receptor (IFNAR) signaling pathway. When cGAMP-mediated STING pathway was modulated by MMAE, the enhanced signaling pathway and immune response via STING signaling are dominant. Thus, the effect of IFNAR signaling could be ignored in this condition. Indeed, MMAE directly promoted the phosphorylation of the TBK1-STING-IRF3 cascade, and the enhancement could be detected after short time drug exposure (less than 2 hours). Additionally, the enhancement effect could only be detected with microtubule destabilizers, but not microtubule stabilizers. These data suggested the effect of MMAE on STING signaling was not caused by antimitotic and cytotoxic effects of MDAs. MMAE augmented cGAMP-mediated activation of the TBK1-STING-IRF3 signal axis in a cGAS-independent manner, which is different from the regulation mechanism of other microtubule stabilizers and destabilizers by promoting the activation of the cGAS-STING pathway through micronucleus formation and mitochondrial DNA release [45,46].

Studies have shown that microtubules are required for efficient intracellular mature virus (IMV) formation and are essential for intracellular enveloped virus (IEV) assembly [38,47]. In particular, the completion of infection cycles of HSV-1, PRV, and VACV is strictly dependent on microtubule movement [27,48]. Accordingly, it’s reasonable to hypothesize that chemicals disrupting microtubule networks could suppress viral infection. For example, nocodazole treatment reduced the number of virus particles by three fold in the cell [27]. Moreover, selective depolymerization of microtubules by nocodazole or colchicine effectively prevents the migration of murine polyomavirus (double stranded DNA virus) toward nucleolus and the exit of progeny virus in cells [35]. These findings are supported by a recent study demonstrating that podofilox, an antiviral drug used typically for the treatment of warts, destroys microtubules and prohibits virus entry following initial binding [49]. Intriguingly, these microtubule destabilizers, including nocodazole, colchicine, and podofilox, were among the compounds that enhanced STING signaling in our high throughput screening assay. Therefore, we systematically compared different microtubule destabilizers and chose MMAE for further study because of its potent antiviral activity and less pronounced cytotoxic effects. While the results suggest that other clinically available MDAs could be investigated for their potential antiviral activities, it would be required to conduct side-by-side experiments to compare different MDAs in future studies.

We revealed that MMAE alone could inhibit viral infection, however the inhibition was independent of STING-mediated activation of IFN pathway and was through viral transport by disrupting the microtubule network (Fig 6G). Remarkably, the combination of MMAE and cGAMP strongly increased antiviral activity. We found that MMAE enhanced cGAMP-STING signaling cascade at low doses (nanomolar level in cellular assay) and exerted excellent broad-spectrum antiviral activities. The combination of MMAE and cGAMP enhanced the production of IFNs *in vitro* and *in vivo*. IFNs are essential components of the immune response against infections, but prolonged exposure to IFNs can lead to suppression of the immune response or hyper-activation [50,51]. Thus, the treatment with MMAE and cGAMP need to be evaluated and controlled carefully to avoid side effects. Moreover, out data showed that MMAE could enhance the NF-kB immune signaling induced by cGAMP-STING pathway. The exact mechanism of STING-mediated NF-kB signaling still need to be revealed, and further studies are required to investigate the mechanism for how MMAE and cGAMP regulates NF-kB signaling. In addition, MMAE showed synergistic effect with not only cGAMP, but also other STING agonists [26], suggesting that the combination of MMAE with other available STING agonists could also have antiviral activities, expanding possible therapeutic options. Collectively, these results support the notion that MMAE, a potent MDA with proven clinical safety at tested concentrations, might have potential to be used as antiviral drug in combination with STING agonists. Given that MMAE is widely used in the treatment of tumor, the combination of MMAE and STING agonists may also have potential in cancer therapy.

Certain HSV-1 strains sensed by the cellular cGAS-STING pathway could induce strong type I IFNs for the host to curtain viral replication [52,53]. Hence, we chose the mouse infection model with HSV-1 to study the antiviral activity of MMAE combined with cGAMP *in vivo* and to verify the effect of MMAE on the innate immune response to DNA virus. Although MMAE alone did not induce IFN response, it generated comparable antiviral effect to that of cGAMP in WT mice by disrupting microtubule networks. In addition, we showed that MMAE augmented cGAMP-mediated STING signaling and thus mediated the production of IFNs and the induction of ISGs *in vivo*, which is the major antiviral effector against HSV-1 infection. Ablation of STING in mice resulted in impaired IFN response and more vulnerable defense against HSV-1 infection. This led to the abolition of the antiviral effect of cGAMP with or without MMAE in *Sting*^*gt/gt*^ mice. These data suggested that STING-mediated IFNs induced by MMAE and cGAMP co-treatment play an essential role in this antiviral response. Further studies are required to characterize which immune cells are activated by the enhanced STING immune signaling and what is the precise role of related IFN expression during this process. Understanding this modulatory network may provide useful information for antiviral treatment.

Our work revealed a new role for microtubules in the regulation of STING-induced immune signaling and antiviral responses. Our results suggested that the combination of MMAE and cGAMP may be a potential strategy in antiviral treatment. The findings open a new possibility to fight against viral outbreaks by combining STING agonists, MDAs or even MDA-conjugated antibodies.

## Materials and methods

### Ethics statement

Animals used in this study were treated in accordance with the guidelines on humane care, and the protocols were approved by the Institutional Animal Care and Use Committee of Tsinghua University.

### Reagents and antibodies

Monomethyl auristatin E (HY-15162), VcMMAE (HY-15575), RO8191 (HY-W063968), Brefeldin A (HY-16592), Bafilomycin A1 (HY-100558), Cycloheximide (HY-12320), Nocodazole (HY-13520), Combretastatin A4 (HY-N2146), Vinblastine sulfate (HY-13780), Ansamitocin P-3 (HY-15739), 4’-Demethylepipodophyllotoxin (4’-DMEP, HY-17435), Colchicine (HY-16569), Albendazole (HY-B0223), Tubulin inhibitor 1 (HY-112607), Avanbulin (HY-106008), SB-216 (HY-144898), Fenbendazole (HY-B0413), Entasobulin (HY-16777), Topotecan (HY-13768), Epothilone B (HY-17029), Etoposide (HY-13629), Paclitaxel (HY-B0015), and Docetaxel (HY-B0011) were purchased from MedChemExpress. HT-DNA (#D6898) and LPS (Lipopolysaccharides from Escherichia Colio0111:B4, No. 297-473-0) were purchased from Sigma-Aldrich. 2’3’-cGAMP (tlrl-nacga23), 3’3’-cGAMP (tlrl-nacga), c-di-GMP (tlrl-nacdg), c-di-AMP (tlrl-nacda), Poly(I:C) (tlrl-picw), and Human/Murine IFN-beta bioluminescent ELISA kit (luex-hifnbv2, luex-mifnbv2) were purchased from InvivoGen. IFNβ protein (10704-HNAS) and full-length clone DNA of human tubulin (TUBA1B, HG14070-CF) were purchased from SinoBiological. CellTiter-Glo Luminescent Cell Viability Assay (G7570) was purchased from Promega. LysoTracker Deep Red (L12492) and ER-Tracker Blue-White DPX (E12353) were purchased from Invitrogen. Anti-GM130 antibody (No. 610823) was purchased from BD Biosciences. Rabbit antibodies against STING (#13647), p-STING (#19781), p-TBK1 (#5483), p-IRF3 (#4947), GAPDH (#2118) were purchased from Cell Signaling Technology. Rabbit antibody against IRF3 (11312-1-AP) was purchased from proteintech. Rabbit antibodies against β-tubulin (PA5068), LC3B (T55992), and mouse antibody against GFP-Tag (M20004) were purchased from Abmart. Rabbit antibody against IFNAR2 (A1769) was purchased from ABclonal. Mouse antibody against HSV-1 gD (sc-21719) was purchased from Santa Cruz Biotechnology. Mouse antibody against enterovirus 71 (GTX630191) was purchased from GeneTex. IL-1β was expressed and purified as described in our previous study.

### Cell culture and transfection

THP1-Lucia ISG cells (No. thpl-isg, InvivoGen) and THP1-Lucia NF-κB cells (No. thpl-nfkb, InvivoGen) were cultured in RPMI-1640 medium (Gibco) supplemented with 10% (v/v) fetal bovine serum (FBS, Gemini), 1% penicillin and streptomycin (100 IU/ml and 100 μg/ml respectively, Solarbio) at 37°C in 5% CO_2_ cell culture incubator. RAW-Lucia ISG cells (No. rawl-isg, InvivoGen), HeLa (CCL-2, ATCC), Vero (CCL-81, ATCC), L929 (CCL-1, ATCC), BJ-5ta (CRL-4001, ATCC), HEK293T (CRL-3216, ATCC), and BMDMs (mouse bone marrow-derived macrophages) cells were cultured in Dulbecco’s Modified Eagle’s Medium (DMEM, Gibco) supplemented with 10% FBS (Gemini), 1% penicillin and streptomycin (100 IU/ml and 100 μg/ml respectively, Solarbio) at 37°C in 5% CO_2_ cell culture incubator. HeLa (hSTING), HeLa (hSTING-GFP), HeLa (mCherry-TUBA1B), HeLa (overexpression of hSTING and mCherry-TUBA1B), THP1-Lucia ISG (STING KO, MyD88 KO, STAT1 KO, STAT2 KO, STAT3 KO), THP1-Lucia ISG (STING KO) overexpressing hSTING or hSTING (S366A) cells were generated by transfection. Human STING and human TUBA1B sequences were cloned into the gateway cloning vector pCDH-CMV-MCS-IRES-puromycin/blasticidin (Addgene) to generate lentivirus expression constructs, and loaded into HEK293T cells. For CRISPR/Cas9 knockout, the sgRNA sequences targeting STING (sgRNA-Forward: 5’-ACTCTTCTGCCGGACACTTG-3’ and reverse 5’-CAAGTGTCCGGCAGAAGAGT-3’), MyD88 KO (sgRNA-Forward: 5’-TCAGAAGCGACTGATCCCCA-3’ and reverse 5’-AGTCTTCGCTGACTAGGGGT-3’), STAT1 (sgRNA-Forward: 5’-CCTGATTAATGATGAACTAG-3’ and reverse 5’-CTAGTTCATCATTAATCAGG-3’), STAT2 (sgRNA-Forward: 5’-GTGGACATTCGACAGTACTT-3’ and reverse 5’- AAGTACTGTCGAATGTCCAC-3’), or STAT3 (sgRNA-Forward: 5’-AGCTACAGCAGCTTGACACA-3’ and reverse 5’-TGTGTCAAGCTGCTGTAGCT-3’), p65 (sgRNA-Forward: 5’- GGCGCTCAGTTTCCAGAACC-3’ and reverse 5’- GGTTCTGGAAACTGAGCGCC-3’), p50 (sgRNA-Forward: 5’- AGAAGTATTTCAACCACAGA-3’ and reverse 5’- TCTGTGGTTGAAATACTTCT-3’) were inserted into plentiCRISPR v2 plasmid (Addgene), and packaged in HEK293T cells. Viral media were harvested after 48 h and 72 h, and passed through a 0.22 μm filter. Target cells were selected with 2 μg/ml puromycin (Beyotime Biotechnology) and 5 μg/ml blasticidin (Beyotime Biotechnology) for 1 week. Resistant cells were analyzed by western blot for establishment of target knockout.

### Primary cell preparation

Mouse Embryonic Fibroblasts (MEFs) were isolated from WT ICR mice pregnant (Beijing Vital River Laboratory Animal Technology Co., Ltd.) for 13.5~14 days. The muscle tissues of embryos were digested overnight with 3 mL 0.25% trypsin-EDTA at 4°C. After repetitive pipetting, digested tissues were filtered through a 70 μm strainer. After wash with PBS, MEFs were cultured in DMEM supplemented with 10% FBS (Gibco), 1% nonessential amino acids (Gibco), 1% GlutaMAX (Gibco), 1% penicillin and streptomycin (100 IU/ml and 100 μg/ml respectively, Solarbio), and 6 μM β-mercaptoethanol (Sigma Aldrich) for subsequent experiments.

For BMDMs preparation, mouse femurs and tibias were dissected from WT, *Sting*^*gt/gt*^, or *Myd88*^*-/-*^ C57BL/6 mice. Bone marrow was flushed out with RPMI-1640, and treated with red blood cell lysis buffer (C3702, Beyotime Biotechnology) for 2 min at room temperature. Debris was filtered through a 70 μm strainer. The remaining cells were differentiated into macrophages (BMDMs) by culturing for 6–7 days in L929 cells-conditioned medium (LCCM, DMEM, 20% FBS, 30% L929 culture medium). The medium was changed every three days. Differentiated BMDMs were used for subsequent experiments.

### Production and infection of viruses

Sendai virus (SeV, a gift from Zhengfan Jiang, Peking University) was propagated in chicken embryos, and allantois fluid containing the virus was harvested 72 h after SeV infection. Herpes simplex virus 1-GFP (HSV-1-GFP, KOS strain, a gift from Dr. Daxing Gao, University of Science and Technology of China), vaccinia virus-GFP (VACV-GFP, VR-2035 strain, a gift from Pro. Haiyan Xie and Pro. Lili Huang, Beijing institute of technology), enterovirus 71 (EV-A71 clinical strain were kindly provided by Guangzhou center for disease control and prevention), and pseudorabies virus (PRV, Bartha strain, a generous gift from Guoyu Yang, Henan Agricultural University) were amplified in Vero cells. Vesicular stomatitis virus-GFP (VSV-GFP, a gift from Qiang Ding, Tsinghua University) was propagated in HEK293T cells. The supernatant containing the virus (HSV-1-GFP, VACV-GFP, EV-A71, VSV-GFP, PRV-GFP) was harvested when cell lysis was observed. After centrifugation at 3000 rpm at 4°C, Viral media was filtered through a 0.22 μm filter. Concentration of viruses using Amicon Ultra-15 Centrifugal Filters (30kDa, Millipore) was an optional step. Virus-containing supernatants were aliquoted and stored at -80°C. For *in vitro* infection, THP1-Lucia ISG (WT, STING KO), L929 (WT, STING KO), HeLa (mCherry-TUBA1B) and Vero cells were infected with HSV-1-GFP (MOI = 1), VSV-GFP (MOI = 0.1), PRV-GFP (MOI = 1) VACV-GFP (MOI = 5), EV-A71 (MOI = 1) and then cultured with cGAMP (0.5 μM) and/or MMAE (0.25 μM) for 24 h or indicated times. The cells were imaged with Olympus IX83 Inverted fluorescence microscope. The fluorescence intensity of virus-GFP was determined by ImageJ software. Expression of viral HSV gD, EV-A71, GFP-tagged VSV, VACV and PRV were detected by western blot.

### Luciferase reporter assay

THP1-Lucia ISG (WT, STING KO, MyD88 KO, STAT1 KO, STAT2 KO,STAT3 KO) and RAW-Lucia ISG cells were seeded in 96-well plates at a density of 3 × 10^6^ cells/ml. Cells were treated with 0.5 μM cGAMP, 1μg LPS, 1μg IL-1β, 10 μg Poly(I:C), HSV-1-GFP (MOI = 1), VSV-GFP (MOI = 0.1), or SeV (MOI = 0.1) with or without indicated doses of MMAE for 24 h. For ISRE reporter activity, 20 μl cell suspension was mixed with 60 μl substrate detection reagent (50 mM HEPES pH 7.0, 50 mM NaCl, 0.05% CHAPS, 10 mM EDTA, and 1 μM Coelenterazine) and added to each well of a 96-well white plate. Luminescence was read using a Cytation Cell Imaging Reader (BioTek), and the fold change of luminescence was normalized to vehicle (DMSO)-treated cells.

### Quantitative RT-PCR analysis

THP1-Lucia ISG cells (WT, STING KO) and BMDMs (WT, *Sing*^*gt/gt*^, *Myd88*^*-/-*^ mice) were seeded in 12-well culture plates, and treated with 0.5 μM cGAMP and/or MMAE (0.5 μM, 1 μM) for 6 h. The total RNA was extracted by M5 HiPer Total RNA Extraction Reagent (MF034-01, Mei5 Biotechnology), and cDNA was synthesized using Quantscript RT Kit (KR103, TIANGEN BIOTECH) according to the manufacturer’s instructions. Real-time PCR was performed using 2× M5 HiPer SYBR Premix EsTaq (MF787, Mei5 Biotechnology) in a CFX96 Real Time System (Bio-Rad) according to manufacturer’s instructions. Amplification cycle of each gene was normalized to β-actin. The primers for human /mouse *IFNβ*, *CXCL10*, *CCL5*, *IL-6*, *ISG15*, *TNFα*, *IFIT3*, *IFITM1*, are included in S1 Table. Customized primers were synthesized by Tsingke Biotechnology Co., Ltd. Relative value of gene expression was calculated by the 2^-ΔCt^ method.

### Measurement of cell viability

The cell viability was represented by the total ATP detected with the CellTiter-Glo Luminescent Cell Viability Assay (G7570, Promega) according to instructions. Briefly, THP1-Lucia ISG cells were infected with VSV-GFP (MOI = 0.1) or HSV-1-GFP (MOI = 1), and then cultured with cGAMP (0.5 μM) and/or MMAE (0.25 μM) for 24 h. Cell suspension was mixed with the same volume of CellTiter-Glo Substrate (50 μl), and added to each well of a 96-well white plate. The luminescence was measured by a Cytatio Cell Imaging Reader (BioTek), and the fold change of luminescence was normalized to DMSO-treated cells.

### Enzyme-linked immunosorbent assay (ELISA)

THP1-Lucia ISG cells (WT, STING KO) and BMDMs (WT, *Sing*^*gt/gt*^, *Myd88*^*-/-*^ mice) were seeded in 12-well culture plates, and treated with 0.5 μM cGAMP and/or MMAE (0.5 μM, 1 μM), or VcMMAE (0.5 μM) for 12 h. The concentration of IFNβ in cell supernatants or brain tissue homogenates were measured per the manufacturer’s instructions (Human/Murine IFN-beta bioluminescent ELISA kit, InvivoGen).

### Immunofluorescence microscopy

HeLa cells stably expressing hSTING-GFP were cultured on confocal dishes (Corning), and stimulated with 8 μM 2’3’-cGAMP, 2 μM 3’3’-cGAMP, 10 μM c-di-AMP (with 1 mg/ml, 1:20,000–1:40,000 perfringolysin O delivery), 2 μg HT-DNA (with PEI transfection) and/or 1 μM MMAE or VcMMAE for 2 h or 8 h (HT-DNA). Stimulated cells were stained with LysoTracker Deep Red (Invitrogen) and ER-Tracker Blue-White DPX (Invitrogen) for 30 min at 37°C. Time-lapse live cell imaging was acquired on a Nikon AX super-resolution confocal microscope using a 100× (NA 1.45) objective and processed in NIS-Elements software. Structured illumination microscope (3D-SIM) imaging was acquired using the Highly Intelligent and Sensitive SIM (HIS-SIM) of Guangzhou Computational Super-resolution Biotech Co., Ltd, and Wiener deconvolution was used in reconstructed images. For immunostaining experiments, HeLa cells (hSTING-GFP) grown on glass coverslips were stimulated as indicated above, and then fixed with 4% paraformaldehyde for 15 min at room temperature, permeabilized with 0.1% Triton X-100 in phosphate-buffered saline (PBS) for 5 min at room temperature. Cells were blocked with 10% goat serum (Gibco) for 1 h at room temperature, and incubated with primary antibodies (Rabbit anti-tubulin, 1:250; Mouse anti-GM130, 1:500) in 3% Bovine Serum Albumin (BSA), 0.25% Tween 20 (PBST) for overnight at 4°C. At last, cells were incubated with respective Alexa Fluor secondary antibodies (Thermo Fisher Scientific) in 3% Bovine Serum Albumin (BSA), 0.25% Tween 20 (PBST) for 2 h, and 4′,6-diamidino-2-phenylindole (DAPI) in PBST for 10 min at room temperature. Immunofluorescence images were imaged and analyzed by Zeiss LSM980 Airyscan2 Confocal microscope using a 63× (NA 1.45) objective.

### LC-MS/MS quantification of cGAMP

THP1-Lucia ISG cells were treated with cGAMP (0.5 μM) for 4 h with or without MMAE (0.5 μM). Cells were lysed in 500 μl-1 ml lysis buffer containing 80% of analytical pure methanol and 2% of pre-chilled acetic acid, and subjected to freeze-thaw cycles with liquid nitrogen. The supernatant was evaporated to dryness, and the extracted cGAMP was reconstituted in 60 μl of water, followed by centrifugation at 16,000 *g* for 15 min at 4°C. The supernatant was collected for LC-MS/MS analysis. The LC-MS/MS analysis was performed on an ACQUITY UPLC I-Class (Waters, USA) coupled to an AB Sciex 6500 Triple Quad mass spectrometer (AB Sciex, USA) with the electrospray ionization (ESI) source. The purchased cGAMP compound (InvivoGen) was used as the standard. Optimized ion transitions (m/z: 675.1–524.1, 675.1–506.1) were used for quantification.

### Western blot analysis

Activation of the STING pathway was assessed by western blot to analyze phosphorylation status of STING, TBK1, and IRF3 using commercially available antibodies. Cells were lysed in lysis buffer (20 mM Tris-HCl pH 7.4, 150 mM NaCl, 10% glycerol, 1% Triton X-100, 0.1% SDS, 1 mM EDTA, 1% Sodium Deoxycholate, 1 mM Na_3_VO_4_, 25 mM β-glycerol-phosphate) supplemented with 0.1 mM PMSF (Beyotime Biotechnology) and 0.5 mg/ml Leupeptin (Solarbio) on ice for 30 min. Lysates were centrifuged at 13,000 rpm for 15 min at 4°C, and the soluble fraction was transferred to a new tube. Protein concentration was determined by the absorbance of 280 nm on NanoDrop One (Thermo Fisher Scientific), and the protein sample was boiled with SDS loading buffer at 95°C for 5 min. Proteins were separated on 12% SDS-PAGE gels, immunoblotted onto nitrocellulose membranes, and subsequently incubated with different primary antibodies overnight. After incubation with HRP-labelled secondary antibodies (Huaxingbio) for 1 h at room temperature, the proteins were detected using ECL substrates (Mei5 Biotechnology).

### Analyses of STING polymers in THP1 cells

THP1-Lucia ISG cells were treated with cGAMP (0.5 μM) for 4 h with or without MMAE (0.5 μM) or VcMMAE (0.5 μM). Cells were collected and resuspended in a buffer containing 25 mM Tris pH 7.5, 5 mM MgCl_2_, 1 mM DTT, 1 mM PMSF, and 0.5 mg/ml Leupeptin on ice for 15 min and sonicated. STING was extracted by a suspension buffer containing 1% NP40, and debris was removed by centrifugation at 800 *g* for 5 min. Supernatants were then mixed with 5× native loading buffer (20 mM Tris pH 7.5, 5 mM MgCl_2_, 150 mM NaCl, 1% NP40, 10% glycerol, 5 mM Na_3_VO_4_, 1 mM DTT, and 0.5 mg/ml Leupeptin) and subjected to native PAGE. STING and p-STING polymers was visualized by western blotting.

### Mice

All mice used in this study were on C57BL/6 background. The C57BL/6 mice were maintained under the specific pathogen-free (SPF) conditions in a barrier-sustained facility and provided with sterile food and water. All experiments were conducted with sex and age-matched mice. WT mice were purchased and bred in the laboratory Animal Resources Center, Tsinghua University. *Sting*^*gt/gt*^ and *Myd88*^*-/-*^ mice were kindly provided by Juanjuan Du lad and Yonghui Zhang lab (Tsinghua University), respectively.

### Viral infection in mice

Age and sex-matched C57BL/6 mice (WT and *Sting*^*gt/gt*^) were treated with PBS, cGAMP (30 μg/mice), MMAE (0.5 mg/kg), or cGAMP along with MMAE by intraperitoneal injection (i.p.) for 2 h. Then, the mice were infected with HSV-1-GFP at 2 × 10^8^ pfu per WT mouse or at 1 × 10^7^ pfu per *Sting*^*gt/gt*^ mouse via intravenous (i.v.) injection and monitored daily on weight loss, body condition score [52], and survival. In a separate experiment (at 2 × 10^8^ pfu per WT mouse or at 1 × 10^7^ pfu per *Sting*^*gt/gt*^ mouse via i.v. injection), mouse brains and spleens were harvested and weighed at day 6 post infection for further analysis. Mouse tissues were crushed using a tissue homogenizer, and freeze-thawed three times to release the virus. Virus titers were detected by qRT-PCR assay. The expressions of viral genes were determined by western blotting and real-time Quantitative PCR. Primer pairs of HSV-1-GFP gene amplification are shown in S1 Table.

For therapeutic of settings, age and sex-matched C57BL/6 mice (WT) were infected with HSV-1-GFP at 1 × 10^7^ pfu (sublethal dosage) per mouse via i.v. injection for 16 h. Mice were treated with PBS, cGAMP (30 μg/mice), MMAE (0.5 mg/kg), or cGAMP along with MMAE by intraperitoneal injection (i.p.) for 3 days. Mouse livers and spleens were harvested and weighed at day 4 post infection for further analysis.

### Statistical analysis

GraphPad Prism 8.0 software was used for data analysis. Data are shown as means ± SEM. Statistical significance was determined by unpaired t-tests for two groups. Multiple comparisons were analyzed by one-way ANOVA with Tukey’s posttests. The survival among different treatment groups was analyzed using the log-rank (Mantel-Cox) test. A value of *p* < 0.05 was considered statistically significant. Significance was defined as n.s. (non-significant, *p* > 0.05), *p < 0.05, **p < 0.01, ***p < 0.001, ****p < 0.0001.

## Supporting information

S1 Fig Only microtubule destabilizers boosted cGAMP-meditated immune responses in THP1-Lucia ISG cells. (A-D) THP1-Lucia ISG cells were treated with cGAMP, or cGAMP plus various microtubule destabilizers (1 μM), microtubule stabilizers (1 μM epothilone B, paclitaxel and docetaxel), or DNA topoisomerase inhibitors (1 μM etoposide and topotecan) for 24 h (A and B) or 6 h (C and D), and the fold changes in luminescent signals were normalized to cGAMP-treated cells (A and B). Immunoblotting was carried out to examine the phosphorylation levels of indicated proteins, and the results are representative of three independent biological replicates (C and D).
(TIF)

S2 Fig MMAE specifically increased cGAMP-mediated immune response in a STING-dependent manner. (A) THP1-Lucia ISG cells (WT and STING KO) were treated with cGAMP and/or MMAE for 6 h, respectively. Total RNA was harvested and *IFNβ*, *CXCL10*, *CCL5*, *TNFα*, *IL-6*, *ISG15*, *IFIT3* and *IFITM1* mRNA expression was measured by real-time PCR (n = 3 biological replicates). (B and C) THP1-Lucia ISG cells (WT and MyD88 KO) were stimulated with cGAMP with or without MMAE for 24 h (B) or 6 h (C), and luciferase signals were measured (B). Phosphorylation of STING downstream signal transduction was assessed by immunoblotting with indicated antibodies (C). (D-G) THP1-Lucia ISG cells were stimulated with cGAMP, Sendai virus (SeV, MOI = 0.1), LPS (1 μg), Poly(I:C) (10 μg) or co-treated with indicated concentrations of MMAE for 24 h. ISRE reporter activity was measured and the fold changes in luminescent signals were normalized to DMSO-treated cells. (H and I) THP1-Lucia NF-kB cells were treated with LPS (1 μg), IL-1β (1 μg) or combined with MMAE for 24 h. Fold changes in NF-kB activation were measured by Lucia luciferase signal and normalized to DMSO-treated cells. Bars are the mean ± SEM of indicated (n) independent experiments. Significance was determined by one-way ANOVA; *p < 0.05, **p < 0.01, ***p < 0.001, ****p < 0.0001, n.s. means non-significant.
(TIF)

S3 Fig MMAE directly enhanced the cGAMP-STING signaling pathway. (A) A model showing whether the potentiation effect of MMAE is dependent on the direct STING-IRF3 signal axis or the indirect IFNα/β and its receptors (IFNAR) pathway. (B-I) ISRE reporter activities and STING phosphorylation cascades were measured in response to cGAMP, RO8191 (0.25 μM, an IFNAR2 agonist) or combined with indicated MMAE for 24 h or 6 h in THP1-Lucia ISG cells (WT, STAT1 KO, STAT2 KO and STAT3 KO). The fold changes in luminescent signals were normalized to DMSO-treated cells. The activation of STING signaling was assessed by immunoblotting. Data are representative of three independent experiments. Bars are the mean ± SEM of indicated (n) independent experiments. Significance was determined by one-way ANOVA; *p < 0.05, **p < 0.01, ***p < 0.001, ****p < 0.0001, n.s. means non-significant.
(TIF)

S4 Fig MMAE amplified the STING signaling cascade by increasing the number of STING puncta induced by CDNs. (A) Chemical structure of VcMMAE (valine-citrulline (Vc) conjugate to MMAE, a part of ADC). (B) HeLa cells stably expressing hSTING-GFP were treated with 3’3’-cGAMP (2 μM), cyclic-di-AMP (10 μM), HT-DNA (2 μg, transfection with PEI) with or without MMAE (1 μM) for 2 h or 8 h (HT-DNA) in the presence or absence of brefeldin A (BFA 1μM), followed by confocal imaging. Green, STING-GFP. Nuclei were stained with 4’,6-diamidino-2-phenylindole (DAPI; blue). Scale bars, 10 μm. (C and D) THP1-Lucia ISG cells were stimulated with cGAMP (0.5 μM) for 24 h (C) or 6 h (D) in the presence or absence of MMAE (indicated doses or 1 μM) or VcMMAE (indicated doses or 1 μM). Fold changes in luminescent signals were normalized to cGAMP-treated cells (C). The induction of *CXCL10*, *CCL5*, *IL-6*, *TNFα*, *IFITM1*, and *IFIT3* expression was analyzed by real-time PCR (D). Data are presented as mean ± SEM and analyzed by one-way ANOVA (*p < 0.05, **p < 0.01, ***p < 0.001, ****p < 0.0001, n.s. means non-significant).
(TIF)

S5 Fig Multiple microtubule destabilizers altered STING trafficking pattern during cGAMP-mediated STING activation. (A) Fluorescent micrograph shows hSTING-GFP vesicle trafficking in HeLa cells. Time-lapse live cell microscopy recording was started 0 min after cGAMP (8 μM) or co-stimulated with MMAE (1 μM) or VcMMAE (1 μM). Selected frames from the movie are shown in A. Scale bars, 5 μm. (B) HeLa cells (hSTING-GFP) were stimulated with cGAMP (8 μM) with or without various microtubule destabilizers and a microtubule stabilizer (paclitaxel, 1 μM) after 2 h, fixed, permeabilized, and stained for tubulin (red). Nuclei were stained with DAPI (blue). Scale bars, 10 μm. (C) Immunoblotting analysis of STING degradation in THP1 cells treated with cGAMP (8 μM) with or without MMAE (1 μM) in the absence or presence of cycloheximide (CHX, 50 μg/ml) for indicated times. Total STING protein was quantified by image J software (n = 3 biological replicates).
(TIF)

S6 Fig MMAE enhanced cGAMP-mediated antiviral activity *in vitro*. (A-C and E) THP1 cells (WT, STING KO) and L929 cells (WT, STING KO) were infected with VSV-GFP (MOI = 0.1) and HSV-1-GFP (MOI = 1) respectively, and then cultured cGAMP (0.5 μM) and/or MMAE (0.25 μM) for 24 h. The cells were imaged with Olympus IX83 Inverted fluorescence microscope. The fluorescence intensity of viral GFP was determined by ImageJ software, shown on the right of each row of images (n = 15, biological replicates). Scale bars, 100 μm. (D) STING protein were analyzed by immunoblotting in L929 cells (WT and STING KO). (F) L929 cells (STING KO) were infected with HSV-1-GFP (MOI = 1), and then cultured cGAMP (0.5 μM) or MMAE (0.25 μM) for indicated times. Viral propagation was determined by western blot. The results are representative of three independent biological replicates. Bars are the mean ± SEM. Significance was determined by one-way ANOVA; *p < 0.05, **p < 0.01, ***p < 0.001, ****p < 0.0001, n.s. means non-significant.
(TIF)

S7 Fig MMAE synergized with cGAMP-mediated STING signaling to show potent and broad antiviral activity *in vitro*. (A and C) THP1 and L929 cells were infected with VSV-GFP (MOI = 0.1), HSV-1-GFP (MOI = 1) or PRV-GFP (MOI = 1), and then cultured cGAMP (0.5 μM) and/or MMAE (0.25 μM) for indicated times. Viral propagation was determined by western blot. The results are representative of three independent biological replicates. (B) THP1 cells viability was determined by ATP assay after indicated treatments for 24 h. (D) Donor THP1 cells were treated with cGAMP and/or MMAE for 6h, then washed out to produce 24 h-conditioned media, which was added to recipient Vero cells infected or uninfected with VACV-GFP (MOI = 5) or EV-A71 (MOI = 1). Whole-cell lysates were subjected to immunoblotting with specific antibodies at indicated times.
(TIF)

S8 Fig MMAE enhanced the antiviral effects of cGAMP in a STING-dependent manner *in vivo*. (A-G and H-J) WT and *Sting*^*gt/gt*^ C57BL/6 mice (n = 10) were treated with PBS, cGAMP (30 μg/mice), MMAE (0.5 mg/kg), or cGAMP along with MMAE by intraperitoneal injection (i.p.) for 2 h. Then, the mice were infected intravenously with HSV-1-GFP at 2 × 10^8^ pfu per WT mouse or at 1 × 10^7^ pfu per *Sting*^*gt/gt*^ mouse. (A-C and I) Body weight of mice were observed and recorded daily. (D-G, H and J) Six days after virus infection, three C57BL/6 mice (WT and *Sting*^*gt/gt*^) were randomly selected for subsequent experiments. The viral titers of mouse spleens were measured by qRT-PCR assay (n = 3 biological replicates) (D). Expressions of viral genes in spleens were measured by immunoblotting and qPCR analysis (n = 3) (E, F, and J). Expressions of IFNβ and ISGs in spleens were analyzed by qPCR analysis (n = 3) (G and H). (K-P) C57BL/6 mice (WT, n = 4) were infected intravenously with HSV-1 at 1 × 10^7^ pfu per mouse. 16 hours later, the mice were treated with PBS, cGAMP (30 μg/mice), MMAE (0.5 mg/kg), or cGAMP along with MMAE by intraperitoneal injection (i.p.) for 3 days. The viral titers of mouse livers and spleens were measured by qRT-PCR assay (K and N). Expressions of viral genes in livers and spleens were qualified by immunoblotting and qPCR analysis (L, M, O and P).
(TIF)

S1 Movie STING-GFP trafficking. Related to S5A Fig. STING in green. Recording begins at 0 min after stimulation of HeLa cells (STING-GFP). Images were acquired every 15 seconds. Movie shows 2D view in thirty frames (2 hours of live cell movement) per second.
(AVI)

S1 Table Primers used for real-time PCR in this study. (DOCX)
