# Supplementary figures and images for: Microtubule disruption synergizes with STING signaling to show potent and broad-spectrum antiviral activity
Source: PLoS Pathog. 2024 Feb 26;20(2):e1012048. doi: 10.1371/journal.ppat.1012048 (PMC10919859; doi:10.1371/journal.ppat.1012048)

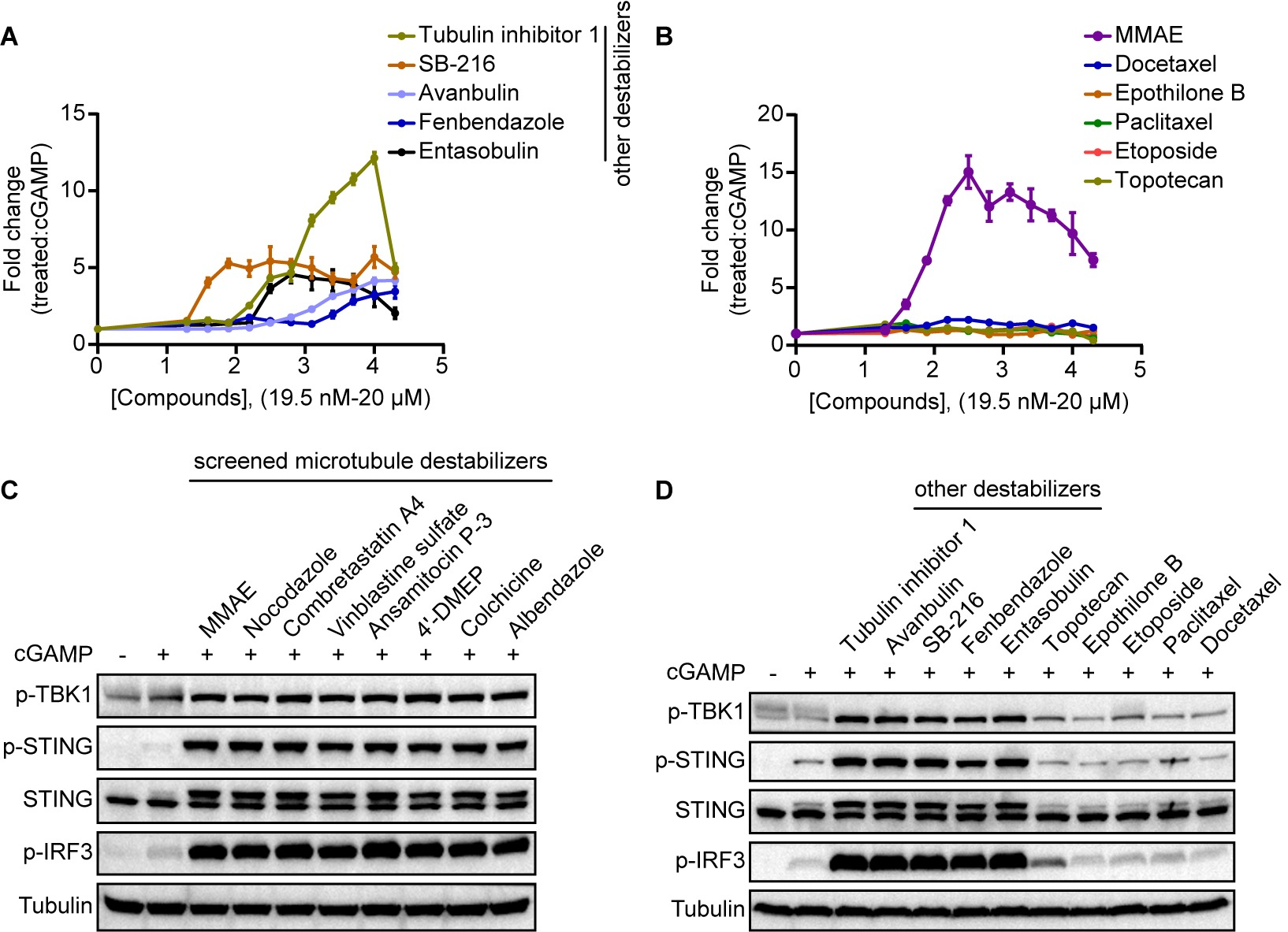

Supplement: S1 Fig — (A-D) THP1-Lucia ISG cells were treated with cGAMP, or cGAMP plus various microtubule destabilizers (1 μM), microtubule stabilizers (1 μM epothilone B, paclitaxel and docetaxel), or DNA topoisomerase inhibitors (1 μM etoposide and topotecan) for 24 h (A and B) or 6 h (C and D), and the fold changes in luminescent signals were normalized to cGAMP-treated cells (A and B). Immunoblotting was carried out to examine the phosphorylation levels of indicated proteins, and the results are representative of three independent biological replicates (C and D). (TIF) [file ppat.1012048.s001.tif]

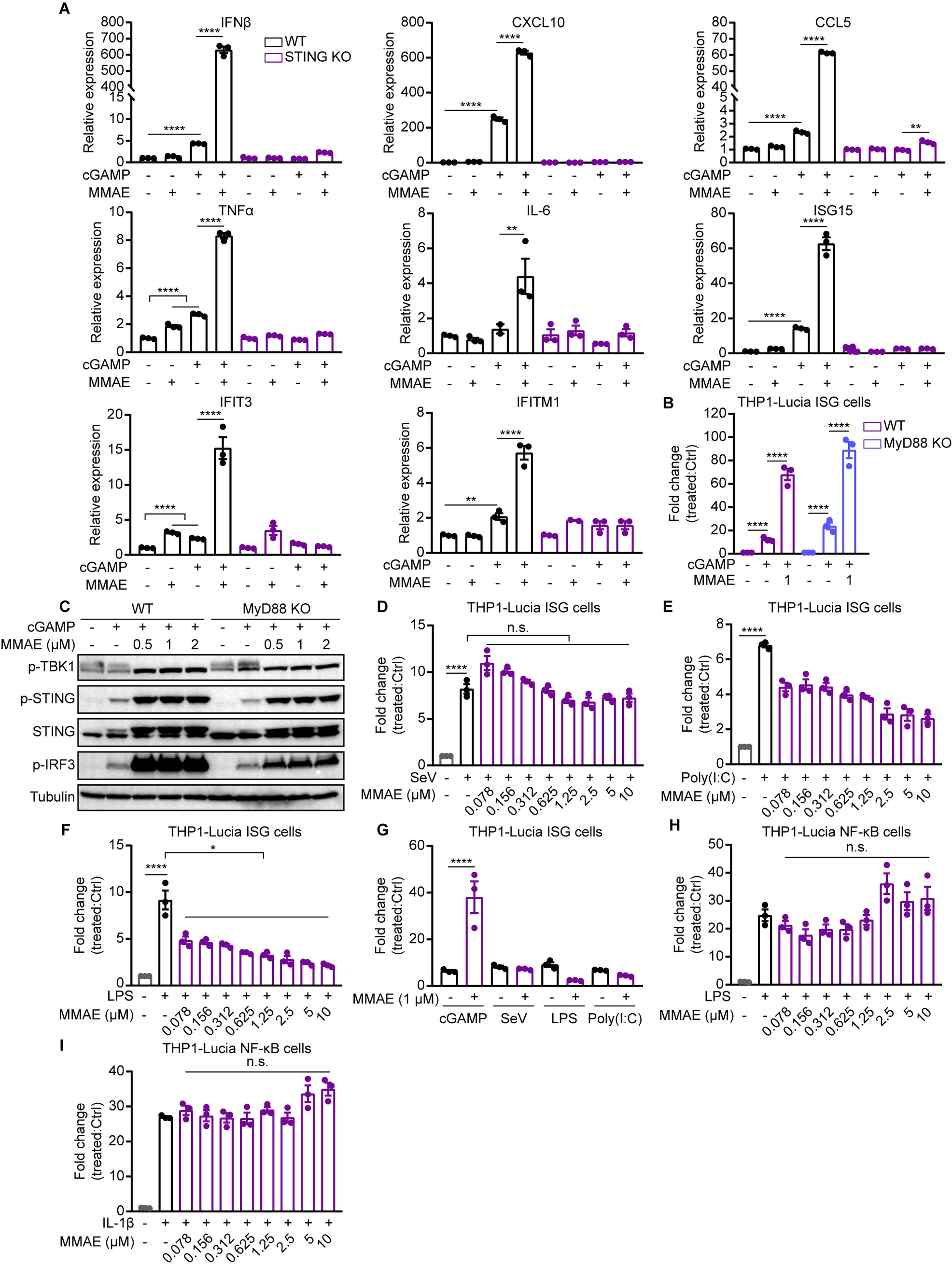

Supplement: S2 Fig — (A) THP1-Lucia ISG cells (WT and STING KO) were treated with cGAMP and/or MMAE for 6 h, respectively. Total RNA was harvested and IFNβ, CXCL10, CCL5, TNFα, IL-6, ISG15, IFIT3 and IFITM1 mRNA expression was measured by real-time PCR (n = 3 biological replicates). (B and C) THP1-Lucia ISG cells (WT and MyD88 KO) were stimulated with cGAMP with or without MMAE for 24 h (B) or 6 h (C), and luciferase signals were measured (B). Phosphorylation of STING downstream signal transduction was assessed by immunoblotting with indicated antibodies (C). (D-G) THP1-Lucia ISG cells were stimulated with cGAMP, Sendai virus (SeV, MOI = 0.1), LPS (1 μg), Poly(I:C) (10 μg) or co-treated with indicated concentrations of MMAE for 24 h. ISRE reporter activity was measured and the fold changes in luminescent signals were normalized to DMSO-treated cells. (H and I) THP1-Lucia NF-kB cells were treated with LPS (1 μg), IL-1β (1 μg) or combined with MMAE for 24 h. Fold changes in NF-kB activation were measured by Lucia luciferase signal and normalized to DMSO-treated cells. Bars are the mean ± SEM of indicated (n) independent experiments. Significance was determined by one-way ANOVA; *p < 0.05, **p < 0.01, ***p < 0.001, ****p < 0.0001, n.s. means non-significant. (TIF) [file ppat.1012048.s002.tif]

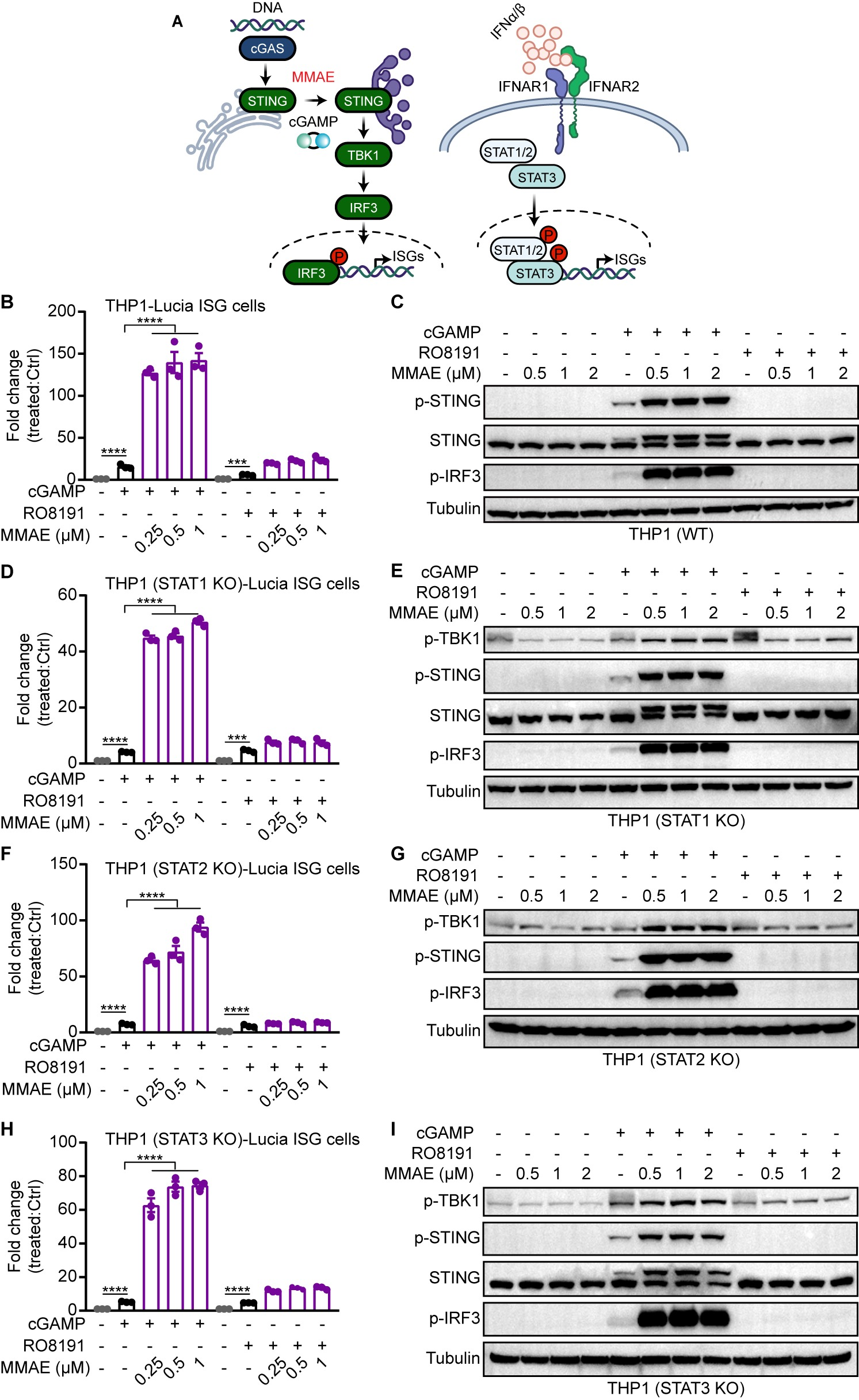

Supplement: S3 Fig — (A) A model showing whether the potentiation effect of MMAE is dependent on the direct STING-IRF3 signal axis or the indirect IFNα/β and its receptors (IFNAR) pathway. (B-I) ISRE reporter activities and STING phosphorylation cascades were measured in response to cGAMP, RO8191 (0.25 μM, an IFNAR2 agonist) or combined with indicated MMAE for 24 h or 6 h in THP1-Lucia ISG cells (WT, STAT1 KO, STAT2 KO and STAT3 KO). The fold changes in luminescent signals were normalized to DMSO-treated cells. The activation of STING signaling was assessed by immunoblotting. Data are representative of three independent experiments. Bars are the mean ± SEM of indicated (n) independent experiments. Significance was determined by one-way ANOVA; *p < 0.05, **p < 0.01, ***p < 0.001, ****p < 0.0001, n.s. means non-significant. (TIF) [file ppat.1012048.s003.tif]

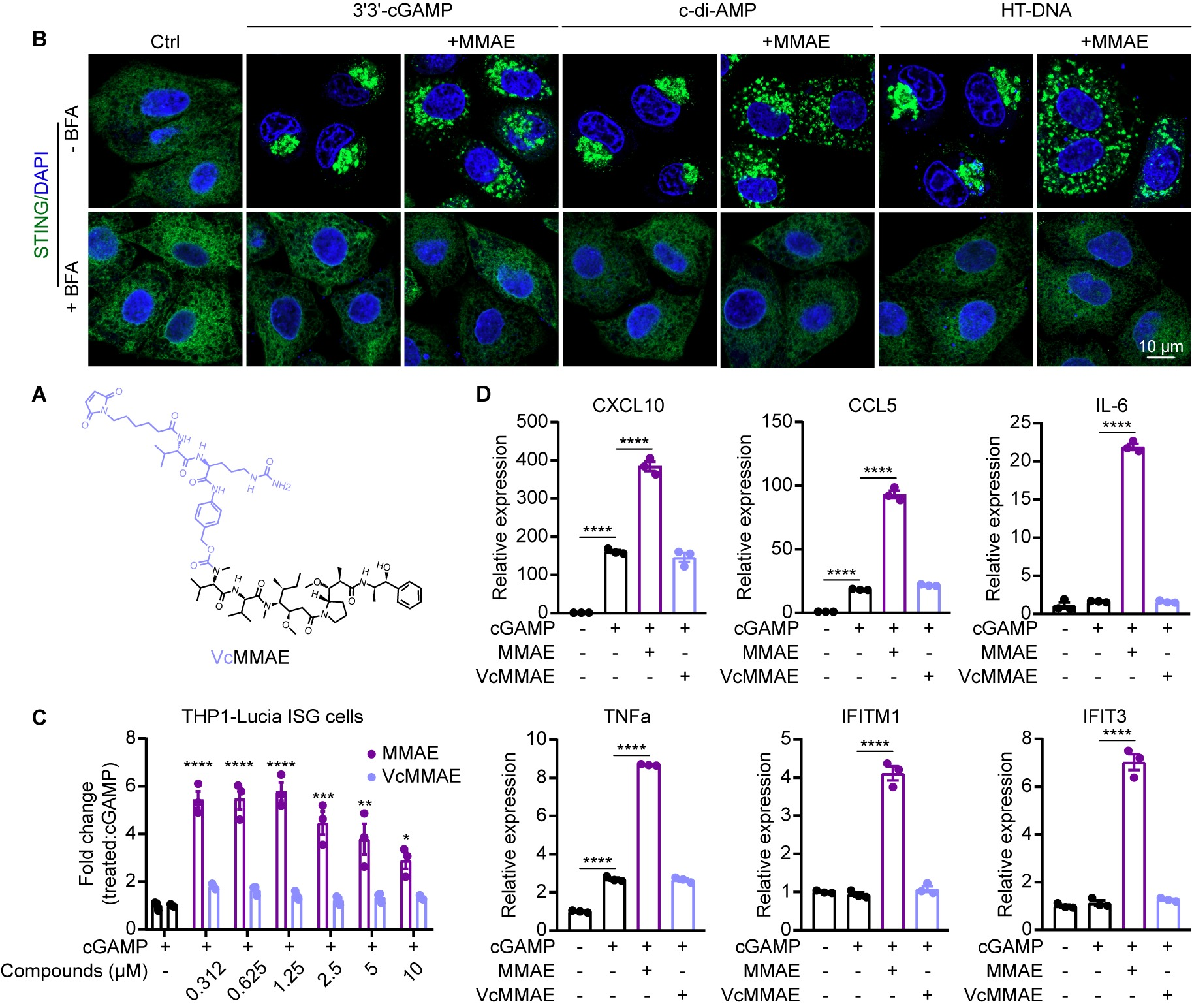

Supplement: S4 Fig — (A) Chemical structure of VcMMAE (valine-citrulline (Vc) conjugate to MMAE, a part of ADC). (B) HeLa cells stably expressing hSTING-GFP were treated with 3’3’-cGAMP (2 μM), cyclic-di-AMP (10 μM), HT-DNA (2 μg, transfection with PEI) with or without MMAE (1 μM) for 2 h or 8 h (HT-DNA) in the presence or absence of brefeldin A (BFA 1μM), followed by confocal imaging. Green, STING-GFP. Nuclei were stained with 4’,6-diamidino-2-phenylindole (DAPI; blue). Scale bars, 10 μm. (C and D) THP1-Lucia ISG cells were stimulated with cGAMP (0.5 μM) for 24 h (C) or 6 h (D) in the presence or absence of MMAE (indicated doses or 1 μM) or VcMMAE (indicated doses or 1 μM). Fold changes in luminescent signals were normalized to cGAMP-treated cells (C). The induction of CXCL10, CCL5, IL-6, TNFα, IFITM1, and IFIT3 expression was analyzed by real-time PCR (D). Data are presented as mean ± SEM and analyzed by one-way ANOVA (*p < 0.05, **p < 0.01, ***p < 0.001, ****p < 0.0001, n.s. means non-significant). (TIF) [file ppat.1012048.s004.tif]

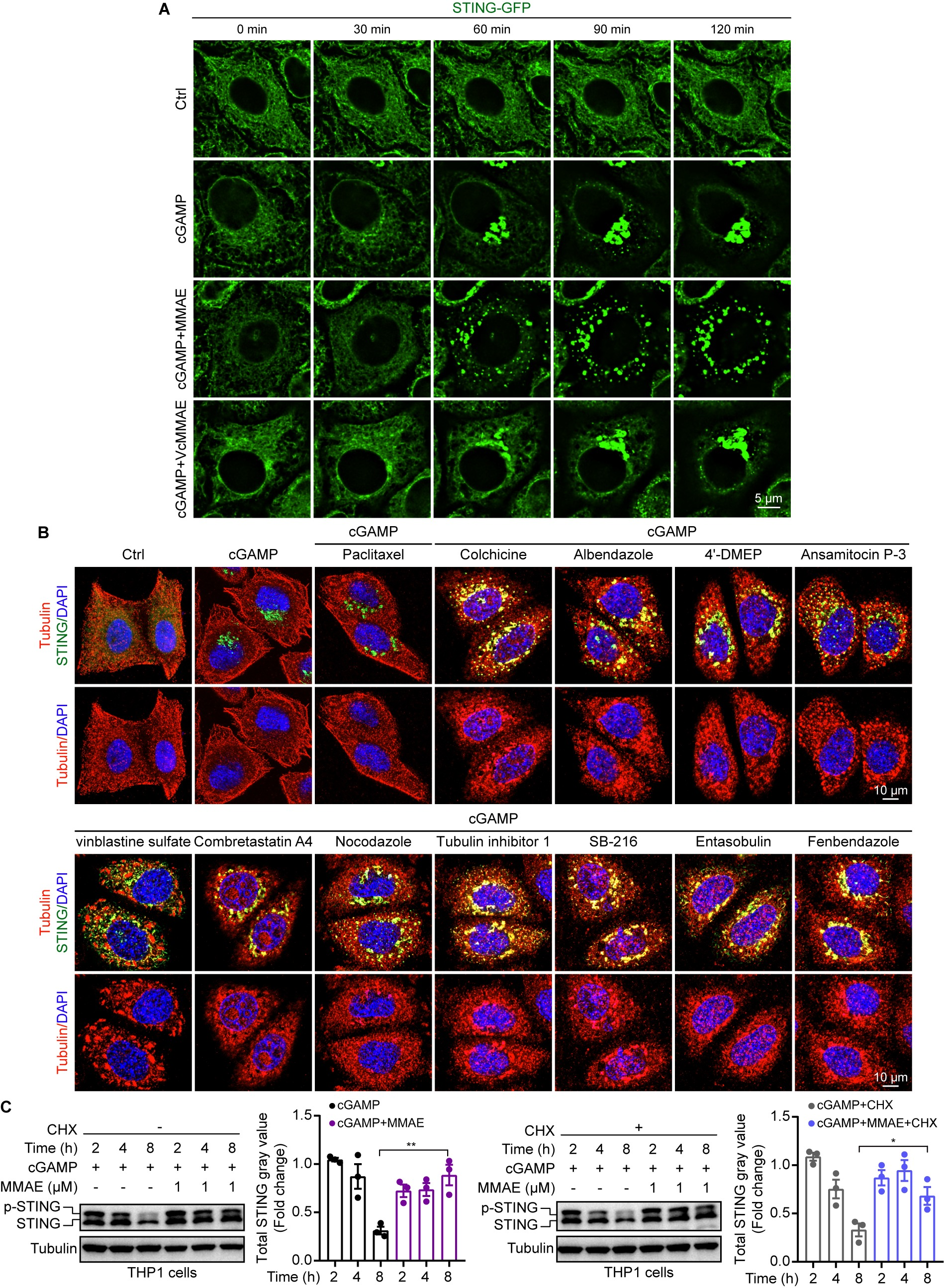

Supplement: S5 Fig — (A) Fluorescent micrograph shows hSTING-GFP vesicle trafficking in HeLa cells. Time-lapse live cell microscopy recording was started 0 min after cGAMP (8 μM) or co-stimulated with MMAE (1 μM) or VcMMAE (1 μM). Selected frames from the movie are shown in A. Scale bars, 5 μm. (B) HeLa cells (hSTING-GFP) were stimulated with cGAMP (8 μM) with or without various microtubule destabilizers and a microtubule stabilizer (paclitaxel, 1 μM) after 2 h, fixed, permeabilized, and stained for tubulin (red). Nuclei were stained with DAPI (blue). Scale bars, 10 μm. (C) Immunoblotting analysis of STING degradation in THP1 cells treated with cGAMP (8 μM) with or without MMAE (1 μM) in the absence or presence of cycloheximide (CHX, 50 μg/ml) for indicated times. Total STING protein was quantified by image J software (n = 3 biological replicates). (TIF) [file ppat.1012048.s005.tif]

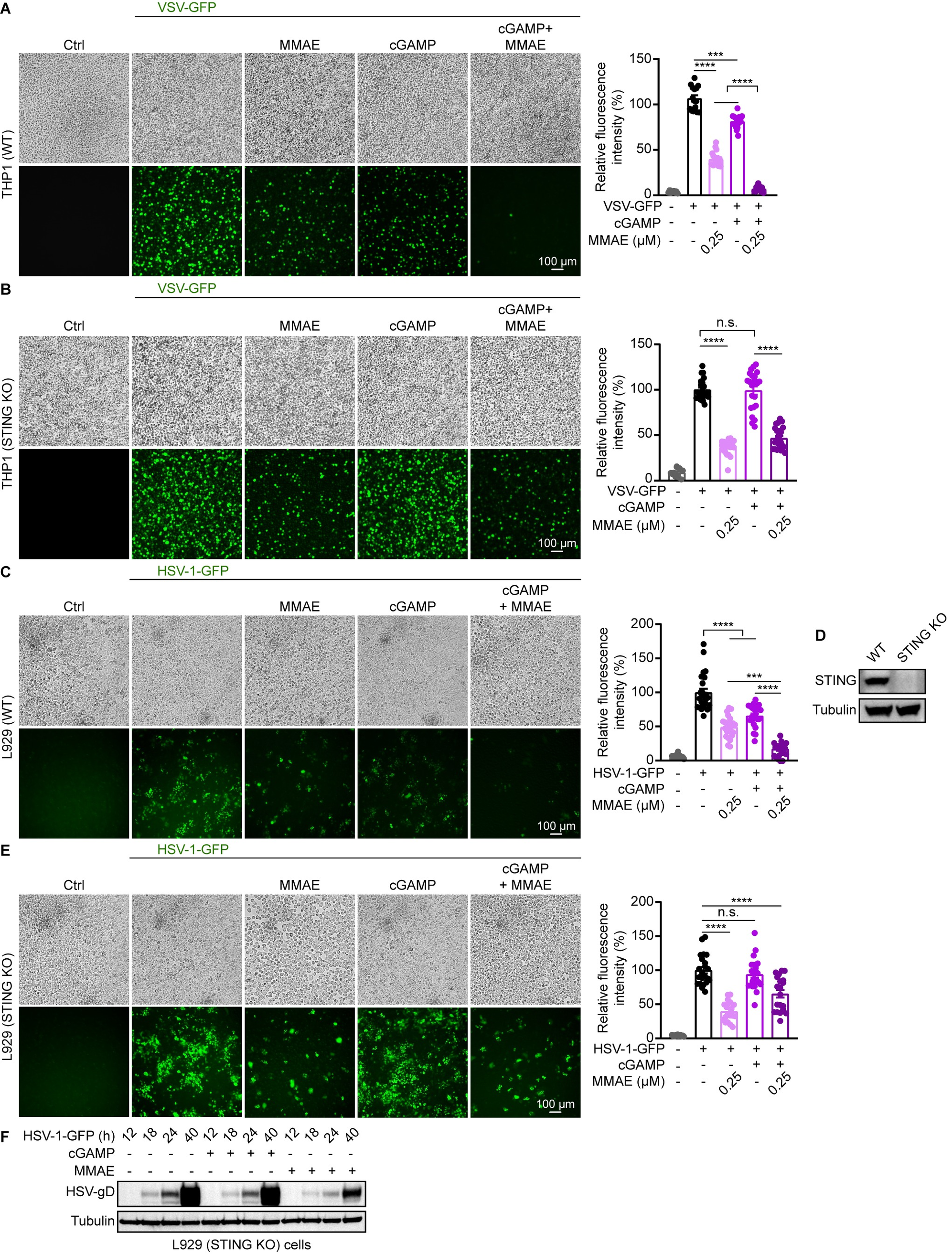

Supplement: S6 Fig — (A-C and E) THP1 cells (WT, STING KO) and L929 cells (WT, STING KO) were infected with VSV-GFP (MOI = 0.1) and HSV-1-GFP (MOI = 1) respectively, and then cultured cGAMP (0.5 μM) and/or MMAE (0.25 μM) for 24 h. The cells were imaged with Olympus IX83 Inverted fluorescence microscope. The fluorescence intensity of viral GFP was determined by ImageJ software, shown on the right of each row of images (n = 15, biological replicates). Scale bars, 100 μm. (D) STING protein were analyzed by immunoblotting in L929 cells (WT and STING KO). (F) L929 cells (STING KO) were infected with HSV-1-GFP (MOI = 1), and then cultured cGAMP (0.5 μM) or MMAE (0.25 μM) for indicated times. Viral propagation was determined by western blot. The results are representative of three independent biological replicates. Bars are the mean ± SEM. Significance was determined by one-way ANOVA; *p < 0.05, **p < 0.01, ***p < 0.001, ****p < 0.0001, n.s. means non-significant. (TIF) [file ppat.1012048.s006.tif]

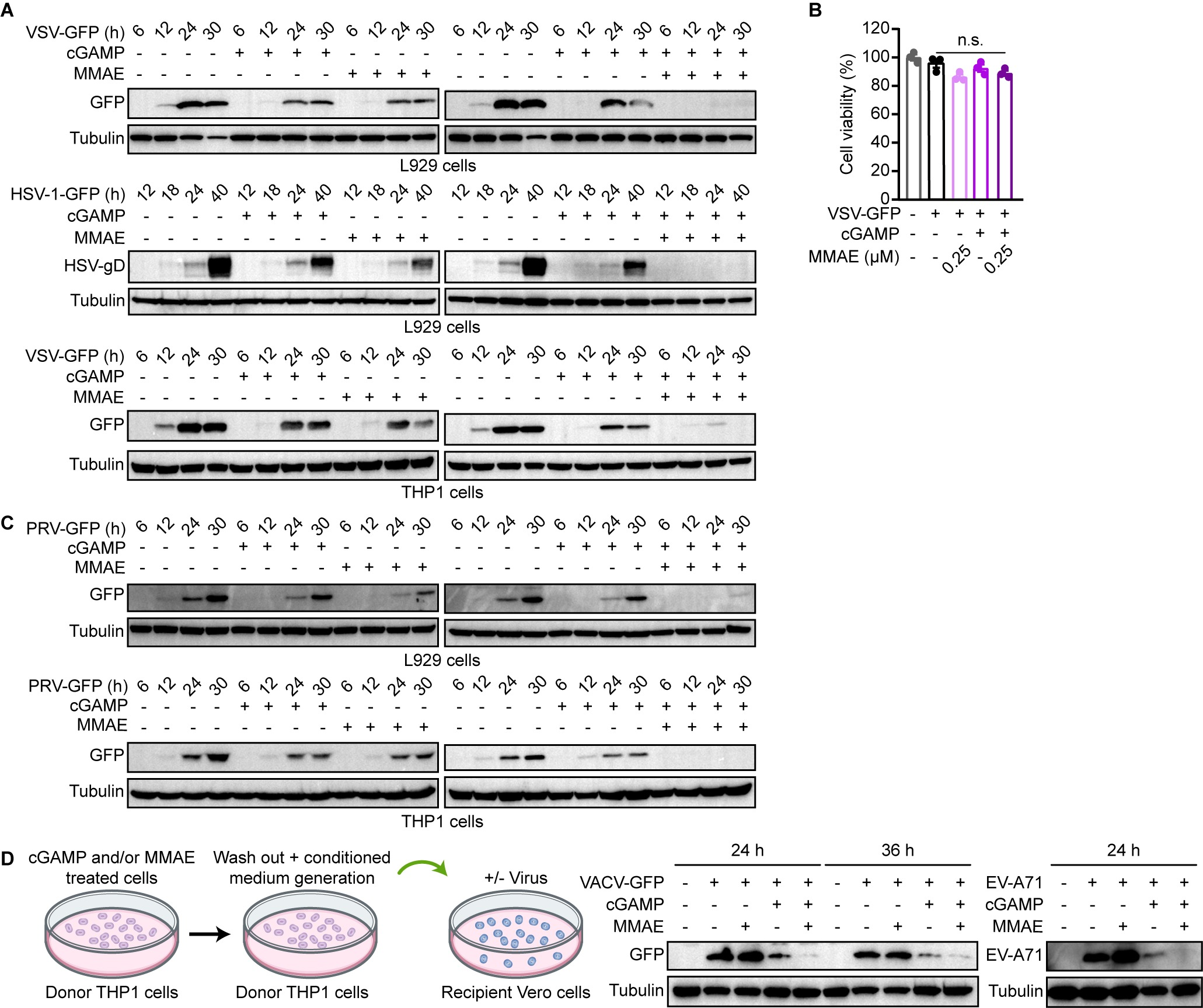

Supplement: S7 Fig — (A and C) THP1 and L929 cells were infected with VSV-GFP (MOI = 0.1), HSV-1-GFP (MOI = 1) or PRV-GFP (MOI = 1), and then cultured cGAMP (0.5 μM) and/or MMAE (0.25 μM) for indicated times. Viral propagation was determined by western blot. The results are representative of three independent biological replicates. (B) THP1 cells viability was determined by ATP assay after indicated treatments for 24 h. (D) Donor THP1 cells were treated with cGAMP and/or MMAE for 6h, then washed out to produce 24 h-conditioned media, which was added to recipient Vero cells infected or uninfected with VACV-GFP (MOI = 5) or EV-A71 (MOI = 1). Whole-cell lysates were subjected to immunoblotting with specific antibodies at indicated times. (TIF) [file ppat.1012048.s007.tif]

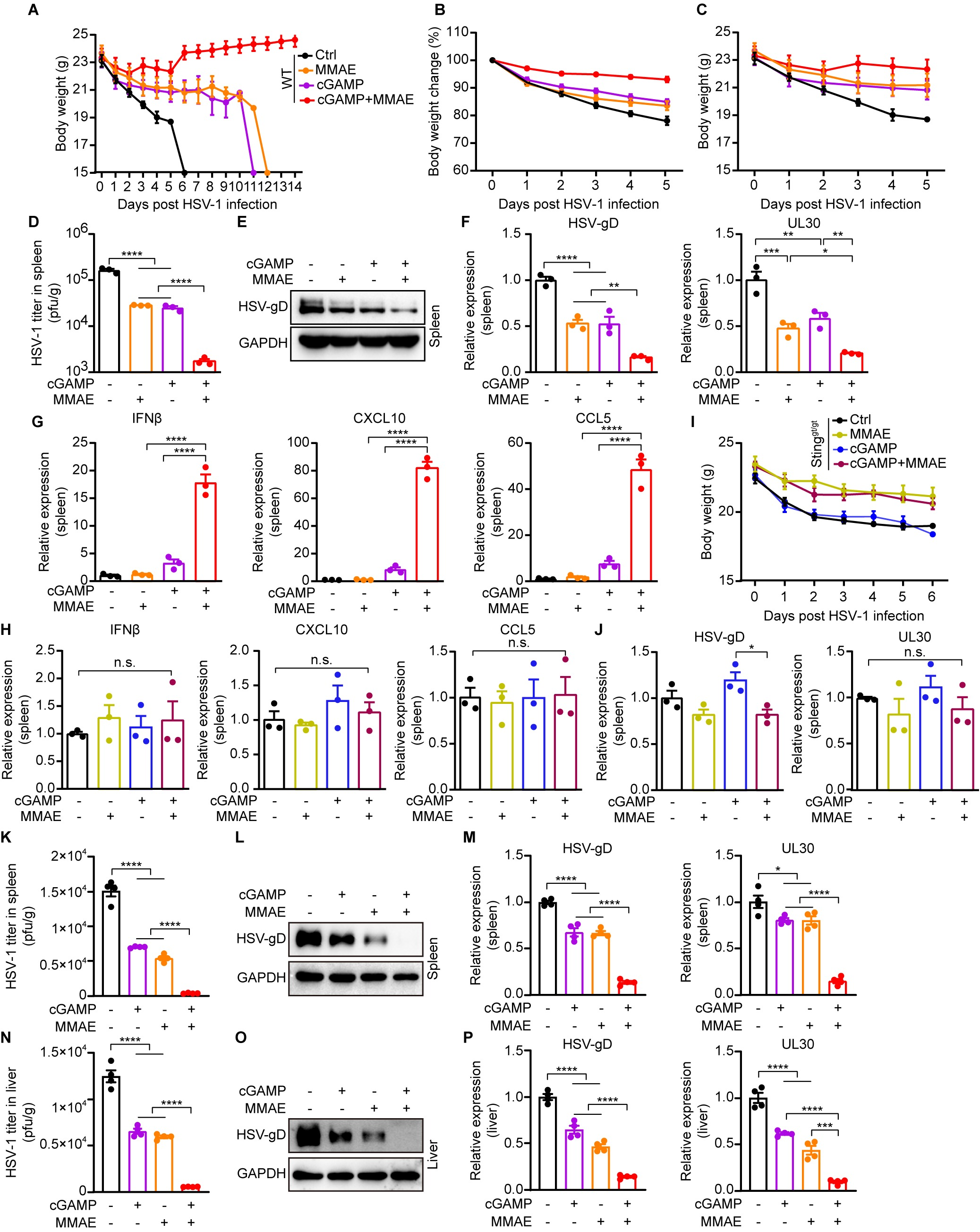

Supplement: S8 Fig — (A-G and H-J) WT and Stinggt/gt C57BL/6 mice (n = 10) were treated with PBS, cGAMP (30 μg/mice), MMAE (0.5 mg/kg), or cGAMP along with MMAE by intraperitoneal injection (i.p.) for 2 h. Then, the mice were infected intravenously with HSV-1-GFP at 2 × 10^8^ pfu per WT mouse or at 1 × 10^7^ pfu per Stinggt/gt mouse. (A-C and I) Body weight of mice were observed and recorded daily. (D-G, H and J) Six days after virus infection, three C57BL/6 mice (WT and Stinggt/gt) were randomly selected for subsequent experiments. The viral titers of mouse spleens were measured by qRT-PCR assay (n = 3 biological replicates) (D). Expressions of viral genes in spleens were measured by immunoblotting and qPCR analysis (n = 3) (E, F, and J). Expressions of IFNβ and ISGs in spleens were analyzed by qPCR analysis (n = 3) (G and H). (K-P) C57BL/6 mice (WT, n = 4) were infected intravenously with HSV-1 at 1 × 10^7^ pfu per mouse. 16 hours later, the mice were treated with PBS, cGAMP (30 μg/mice), MMAE (0.5 mg/kg), or cGAMP along with MMAE by intraperitoneal injection (i.p.) for 3 days. The viral titers of mouse livers and spleens were measured by qRT-PCR assay (K and N). Expressions of viral genes in livers and spleens were qualified by immunoblotting and qPCR analysis (L, M, O and P). (TIF) [file ppat.1012048.s008.tif]
