# Supplementary material for: Microtubule disruption synergizes with STING signaling to show potent and broad-spectrum antiviral activity
Source: PLoS Pathog. 2024 Feb 26;20(2):e1012048. doi: 10.1371/journal.ppat.1012048 (PMC10919859; doi:10.1371/journal.ppat.1012048)
Supplement: S1 Table — (DOCX) [file ppat.1012048.s010.docx]

| **Genes** | **Forward primer** | **Reverse primer** |
| --- | --- | --- |
| human β-actin | CACCATTGGCAATGAGCGGTTC | AGGTCTTTGCGGATGTCCACGT |
| human IFNβ | CTTGGATTCCTACAAAGAAGCAGC | TCCTCCTTCTGGAACTGCTGCA |
| human CXCL10 | GGTGAGAAGAGATGTCTGAATCC | GTCCATCCTTGGAAGCACTGCA |
| human CCL5 | CCTGCTGCTTTGCCTACATTGC | ACACACTTGGCGGTTCTTTCGG |
| human TNFα | CTCTTCTGCCTGCTGCACTTTG | ATGGGCTACAGGCTTGTCACTC |
| human IL-6 | AGACAGCCACTCACCTCTTCAG | TTCTGCCAGTGCCTCTTTGCTG |
| human ISG15 | CTCTGAGCATC CTGGTGAGGAA | AAGGTCAGCCAGAACAGGTCGT |
| human IFITM1 | GGCTTCATAGCATTCGCCTACTC | AGATGTTCAGGCACTTGGCGGT |
| human IFIT3 | CCTGGAATGCTTACGGCAAGCT | GAGCATCTGAGAGTCTGCCCAA |
| mouse β-actin | CATTGCTGACAGGATGCAGAAGG | TGCTGGAAGGTGGACAGTGAGG |
| mouse IFNβ | GCCTTTGCCATCCAAGAGATGC | CACTGTCTGCTGGTGGAGTTC |
| mouse CXCL10 | ATCATCCCTGCGAGCCTATCCT | GACCTTTTTTGGCTAAACGCTTTC |
| mouse CCL5 | CCTGCTGCTTTGCCTACCTCTC | ACACACTTGGCGGTTCCTTCGA |
| HSV-gD | ACGACTGGACGGAGATTACA | GGAGGGCGTACTTACAGGAG |
| UL30 | CATCACCGACCCGGAGAGGGAC | GGGCCAGGCGCTTGTTGGTGTA |
